# Meta-analysis identifies novel risk loci and yields systematic insights into the biology of male-pattern baldness

**DOI:** 10.1038/ncomms14694

**Published:** 2017-03-08

**Authors:** Stefanie Heilmann-Heimbach, Christine Herold, Lara M. Hochfeld, Axel M. Hillmer, Dale R. Nyholt, Julian Hecker, Asif Javed, Elaine G. Y. Chew, Sonali Pechlivanis, Dmitriy Drichel, Xiu Ting Heng, Ricardo C. -H. del Rosario, Heide L. Fier, Ralf Paus, Rico Rueedi, Tessel E. Galesloot, Susanne Moebus, Thomas Anhalt, Shyam Prabhakar, Rui Li, Stavroula Kanoni, George Papanikolaou, Zoltán Kutalik, Panos Deloukas, Michael P. Philpott, Gérard Waeber, Tim D. Spector, Peter Vollenweider, Lambertus A. L. M. Kiemeney, George Dedoussis, J. Brent Richards, Michael Nothnagel, Nicholas G. Martin, Tim Becker, David A. Hinds, Markus M. Nöthen

**Affiliations:** 1 Institute of Human Genetics, University of Bonn, 53127 Bonn, Germany; 2 Department of Genomics, Life & Brain Center, University of Bonn, 53127 Bonn, Germany; 3 German Center for Neurodegenerative Disorders, 53175 Bonn, Germany; 4 Cancer Therapeutics and Stratified Oncology, Genome Institute of Singapore, Singapore 138672, Singapore; 5 TRON-Translational Oncology at the University Medical Center of Johannes Gutenberg University, TRON gGmbH, 55131 Mainz, Germany; 6 Institute of Health and Biomedical Innovation, Queensland University of Technology, GPO Box 2434, Brisbane, Queensland 4001, Australia; 7 Institute of Genomic Mathematics, University of Bonn, 53127 Bonn, Germany; 8 Institute of Medical Informatics, Biometry and Epidemiology, Medical Faculty, University of Duisburg-Essen, 45122 Essen, Germany; 9 Cologne Center for Genomics, University of Cologne, 50931 Cologne, Germany; 10 Computational and Systems Biology, Genome Institute of Singapore, Singapore 138672, Singapore; 11 Department of Biostatistics, Harvard School T.H. Chan of Public Health, Boston, Massachusetts 02115, USA; 12 Dermatology Research Centre, Institute of Inflammation and Repair, University of Manchester, Manchester M13 9PL, UK; 13 Department of Computational Biology, University of Lausanne, 1011 Lausanne, Switzerland; 14 Swiss Institute of Bioinformatics, 1015 Lausanne, Switzerland; 15 Radboud Institute for Health Sciences, Department for Health Evidence, Radboud University Medical Center, 6500 HB Nijmegen, The Netherlands; 16 Centre for Clinical Epidemiology, Departments of Medicine, Genetics and Epidemiology, Lady Davis Institute for Medical Research, Jewish General Hospital, McGill University, Montreal, Quebec, H3T 1E2 Canada; 17 William Harvey Research Institute, Barts and The London School of Medicine and Dentistry, Queen Mary University of London, London EC1M 6BQ, UK; 18 School of Health Sciences and Education, Department of Nutrition and Dietetics, Harokopio University, Attica, 17671 Kallithea, Greece; 19 Institute of Social and Preventive Medicine, University Hospital of Lausanne, 1010 Lausanne, Switzerland; 20 Princess Al-Jawhara Al-Brahim Centre of Excellence in Research of Hereditary Disorders (PACER-HD), King Abdulaziz University, Jeddah 21589, Saudi Arabia; 21 Centre for Cell Biology and Cutaneous Research Barts and The London School of Medicine and Dentistry, Queen Mary University of London, London E1 2AT, UK; 22 Department of Internal Medicine, Lausanne University Hospital (CHUV), 1011 Lausanne, Switzerland; 23 Department of Twin Research & Genetic Epidemiology, King's College London, London SE1 7EH, UK; 24 QIMR Berghofer Medical Research Institute, Brisbane, Queensland 4006, Australia; 25 Institute for Community Medicine, Ernst Moritz Arndt University Greifswald, 17475 Greifswald, Germany; 26 23andMe Inc., Mountain View, California 94040, USA

## Abstract

Male-pattern baldness (MPB) is a common and highly heritable trait characterized by androgen-dependent, progressive hair loss from the scalp. Here, we carry out the largest GWAS meta-analysis of MPB to date, comprising 10,846 early-onset cases and 11,672 controls from eight independent cohorts. We identify 63 MPB-associated loci (*P*<5 × 10^−8^, METAL) of which 23 have not been reported previously. The 63 loci explain ∼39% of the phenotypic variance in MPB and highlight several plausible candidate genes (*FGF5*, *IRF4*, *DKK2*) and pathways (melatonin signalling, adipogenesis) that are likely to be implicated in the key-pathophysiological features of MPB and may represent promising targets for the development of novel therapeutic options. The data provide molecular evidence that rather than being an isolated trait, MPB shares a substantial biological basis with numerous other human phenotypes and may deserve evaluation as an early prognostic marker, for example, for prostate cancer, sudden cardiac arrest and neurodegenerative disorders.

## Abstract (web summary)

Male-pattern baldness is a common condition in which hair is progressively lost from the scalp. Here, the authors find 23 new genetic variants associated with this condition and suggest that it is not an isolated trait but may share an underlying biological basis with various diseases.

Male-pattern baldness (MPB) has a lifetime prevalence of ∼80% in European men and is characterized by a characteristic pattern of progressive hair loss in distinct populations of androgen-dependent scalp hair follicles^1^. Early-onset MPB in particular can exert profound negative effects on quality of life^2^,^3^. While the pathobiology of MPB remains incompletely understood^4^,^5^ and no causal therapy is available, current MBP treatments are of limited efficacy in many patients and even can have severe adverse effects^6^. Therefore, studies that clarify the biological underpinnings of key-pathophysiological features of MPB and enable the identification of novel molecular targets for more effective therapeutic intervention are needed. Here, molecular genetic studies that enable the identification of early causal events and allow differentiation between disease cause and consequence^7^, hold great potential and have already proven to be successful in the identification of contributing risk factors^8^,^9^,^10^,^11^,^12^. However, for the majority of the identified loci, few data are available concerning the manner on how these genetic variants contribute to (i) the strict androgen-dependency of the phenotype; (ii) the restriction of pathophysiological changes to hair follicles in the frontal and vertex scalp regions; (iii) characteristic changes in hair follicle cycling (anagen-shortening/premature catagen entry); and (iv) the gradual (mini-)organ transformation of pigmented terminal hair into unpigmented vellus hair follicles^4^. Given the high heritability of MPB (*h*^2^∼80%; refs 13, 14), and the fact that a significant fraction of the overall heritable risk for MPB still awaits identification, large-scale genetic studies are an important tool to elucidate the molecular basis of MPB and to gain systematic insights into the underlying pathobiology.

Here, we report the results of the largest genome-wide association studies (GWAS) meta-analysis of MPB to date, that comprised a total of 22,518 individuals from eight independent GWAS samples of European descent. The analysis identifies 63 genome-wide significant loci that explain ∼39% of the phenotypic variance in MPB. More than one-third of these loci (*N*=23) have not been reported previously. Our data highlight highly plausible candidate genes and pathways that are likely to contribute to key-pathophysiological characteristics of MPB such as the deregulation of anagen-to-catagen transition (*FGF5*, *EBF1*, *DKK2*, adipogenesis); increased androgen sensitivity (*SRD5A2*, melatonin signalling); and the transformation of pigmented terminal hair into unpigmented vellus hair (*IRF4*). Some of these genes and pathways may represent promising targets for the development of novel therapeutic options. In addition, our data provide molecular evidence that MPB shares a substantial biological basis with numerous other human phenotypes, which may have major implications in terms of the evaluation of MPB as an early prognostic marker for different phenotypes such as prostate cancer, sudden cardiac arrest or neurodegenerative disorders.

## Results

### Meta-analysis

**Figure 1:**
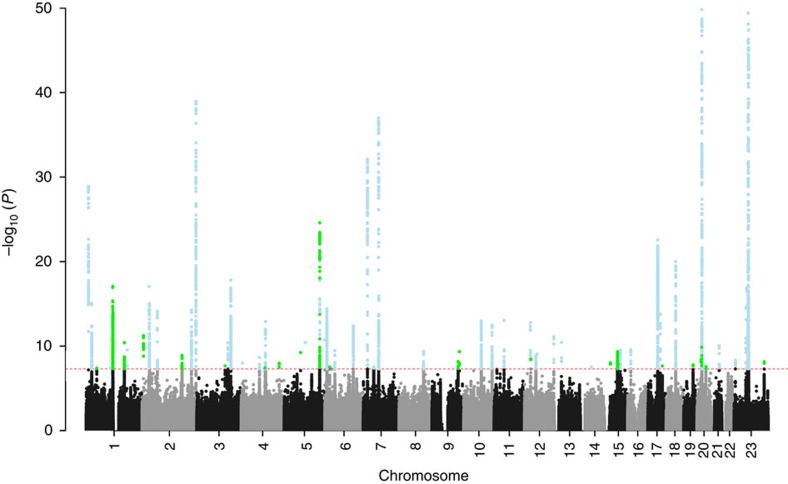
Manhattan plot of association results from the genome-wide meta-analysis. Manhattan plot showing the results of the genome-wide association meta-analysis in 10,846 early-onset MPB cases and 11,672 controls. The *x* axis shows the chromosomal position, and the *y* axis shows the −log_10_ (*P*-value) (METAL) of the association analysis. Previously reported genome-wide significant loci are depicted in blue; novel MPB-risk loci are depicted in green. Data points with *P*<1 × 10^−50^ (METAL) were truncated. The lowest *P*-value was 1 × 10^−320^ (METAL) at the *AR*/*EDA2R*-locus.

Our meta-analysis of eight independent GWAS samples of European descent (10,846 early-onset MPB cases, 11,672 controls; Supplementary Note 1; Supplementary Table 1) identified a total of 63 genome-wide significant MPB-risk loci (*P*<5 × 10^−8^, METAL), which account for ∼39% of the phenotypic variance in MPB (Fig. 1). These include 40 previously reported and 23 novel MPB-risk loci (Table 1; Supplementary Fig. 1). Regarding the genomic context of the 63 genome-wide significant risk loci, the majority of the association peaks (*N*=45, 71%) mapped to intergenic regions. However, 60 (95%) of the association peaks were located within ≤500 kb of a protein coding gene, and a total of 18 (29%) association peaks mapped to intronic or coding regions. Notably, six of the MPB-risk loci are located on the X-chromosome, and may thus contribute to the anecdotal similarity in hair status observed between men and their maternal grandfathers. Among them are the well-established *AR/EDA2R*-locus on Xq12, a locus near *FAM9A* and *FAM9B* on Xp22.31 and two loci near *KLF8* and *TRS2* on Xp11.21. The remaining 57 loci are located on the autosomes. Heritability estimates on the liability scale were 0.14 (±0.03) for the X-chromosome, and 0.34 (±0.12) for the autosomes.

### Risk score analysis

To evaluate the potential value of these association findings for the prediction of MPB risk, a weighted genotype-risk score for MPB was constructed from the lead SNPs of the 63 risk loci. The resulting score was divided into quartiles, and the risk for MPB was tested in each quartile, using the lowest quartile as a reference. As shown in Table 2, an increased risk for MPB was observed across all quartiles, with a substantially increased risk for MPB in quartile four (odds ratio (OR)=4.16, 95% confidence interval (CI)=(2.03–8.55)). This effect was even stronger after adjustment for age (OR=5.14, 95% CI=(2.44–10.86)), which underlines the strong age-dependency of the trait.

### Integration with mRNA and miRNA expression data

To enable biological interpretation of the association findings and to pinpoint plausible candidate genes, the genetic data were integrated with own unpublished data on hair follicle mRNA-, and micro(mi) RNA-expression, an unpublished expression quantitative trait locus (eQTL) dataset from hair follicle (for details see Supplementary Materials and Methods) and two published eQTL data sets from skin and blood^15^,^16^. The comparison with eQTL data sets revealed a colocalization of MPB-risk variants with known regulatory variants (*r*^2^>0.5 between eQTL single nucleotide polymorphism (SNP) and MPB lead SNP) in 25 loci. For 10 of these 25 loci, previous studies have found an association between the MPB lead SNP itself and gene expression for example, of *CRHR1*, which encodes for a receptor for corticotropin releasing hormone (CRH), a known hair growth inhibitor and catagen inductor^17^, *FAM53B* a positive regulator of WNT/beta-catenin-signalling^18^ and other genes, for example, *ANXA2*, *SUCNR1*, *WARS2* that have not yet been associated with hair biology (Supplementary Table 2). Detailed functional follow-up studies are now warranted to confirm these regulatory interactions, and to investigate the contribution of these candidate genes to the development of key MPB pathophysiological signs. A total of 19 association peaks (∼30%) were located within ≤500 kb of a miRNA gene. Eighteen of these 34 miRNA-genes were expressed in human scalp hair follicles, and were predicted to target numerous mRNA genes at MPB-risk loci (Supplementary Table 3). Since miRNAs have been implicated in various aspects of hair biology, such as the control of hair follicle cycling, keratinocyte differentiation/proliferation and melanogenesis^19^, these miRNA-genes and their target genes may constitute plausible candidate genes at these MPB-associated loci.

### DEPICT analysis and enhancer enrichment

**Figure 2:**
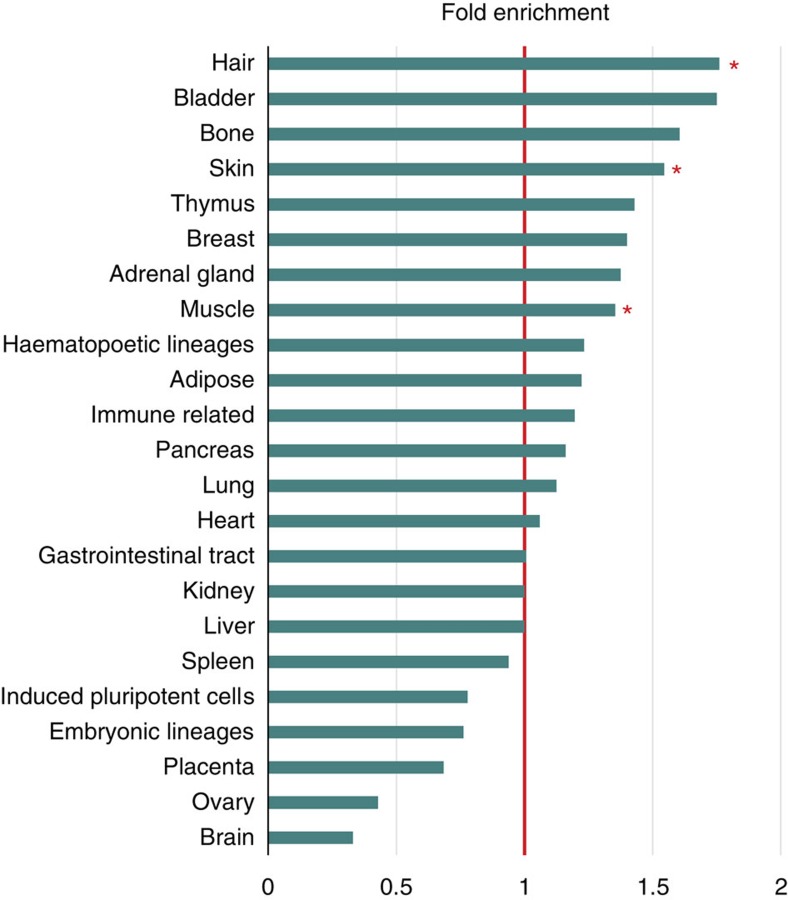
Enrichment of credible SNPs in tissue enhancers. Barplot showing enrichment of MPB credible SNPs across 23 tissue specific enhancer sets. A total of 144 cell lineages and tissues were grouped in 23 categories for this evaluation. The expected number of credible SNPs and the significance of enrichment in each category were estimated in one million enhancer matched permutations, using the rest of the tissue types to define the candidate null set (Supplementary Data 2). Red asterisks indicate tissue types that contain individual cell lineages or tissues that show an enrichment of credible SNPs with an empirical *P*-value of <0.05: non-balding DPCs treated with DHT (hair); one of three foreskin fibroblasts (skin); and one of three psoas muscle lineages.

The DEPICT analysis (Supplementary Tables 4–6) and literature search identified highly plausible candidate genes, such as *FGF5* at 4q21.21 (rs982804; *P*=2.2 × 10^−9^, METAL) and *DKK2* at 4q25 (rs145945174; *P*=1.3 × 10^−13^, METAL). *FGF5* plays an important role in the regulation of anagen-to-catagen transition and the control of human hair length^20^,^21^,^22^. *DKK2* encodes for a member of the family of dickkopf WNT-signalling inhibitors, which are reported to be secreted by dermal papilla cells Fig. f1 Fig. 2(DPCs) in response to androgens and to promote androgen-induced (premature) anagen-to-catagen transition^23^,^24^. Notably, our data indicated a nominally significant enrichment (*P*<0.05, 1 million permutations) of credible MPB SNPs in enhancer regions from DPCs (treated with dihydrotestosterone, DHT), which further supports the hypothesis that DPCs are involved in MPB aetiology^25^ (Fig. 2; Supplementary Data 1). An interesting focus for future research will be to map androgen receptor (AR) binding sites in DPCs and other hair-related cell types to test for enrichment of MPB-risk variants.

Moreover, the strongest MPB-risk allele at 6p25.3 (rs12203592-T; *P*=2 × 10^−11^, METAL) is located within a melanocyte-specific enhancer element, and is reported to have a negative regulatory effect on IRF4 expression. As *IRF4* contributes to the pigmentation of human skin, hair and eye^2^,^26^,^27^, this locus is likely to contribute to the gradual transformation of pigmented terminal hair into unpigmented vellus hair in MPB^28^. In geographical areas distant from the equator, less-pigmented skin, hair and eyes have been under positive selection, presumably due to the fact that this optimizes use of available ultraviolet radiation (UVR) for vitamin D3 generation^29^. Thus, the association between this functional *IRF4* variant and MPB may contribute to the relatively high prevalence of MPB in Europeans.

Another highly plausible candidate gene is located at 2p23.1. Here, the most strongly associated SNP (rs9282858; *P*=8.9 × 10^−18^, METAL) represents a missense variant (c.145G>A; p.Ala49Thr) in *SRD5A2.* This gene encodes for the 5-alpha-reductase type II enzyme, which plays a critical role in androgen metabolism and MBP pathobiology^30^. Interestingly, elevated SRD5A2 levels have been detected in MPB affected scalp, and the SRD5A2 inhibitor finasteride is an effective therapy for MPB^31^,^32^. Additional candidate genes and their biological functions are summarized in Table 1 and Supplementary Data 2.

### Pathway-based analysis

Pathway-based analysis of genes at MPB-risk loci revealed significant enrichment (*P-*value of the right-tailed Fisher's exact test<0.05) in 37 pathways, including the previously implicated androgen metabolism and WNT-signalling pathways (Supplementary Table 7). In addition to these known pathways, our data provide the first genetic evidence for the involvement of additional hormonal pathways such as epidermal growth factor (EGF)-signalling and oestrogen biosynthesis in MPB^5^,^8^,^33^,^34^,^35^,^36^. Enrichment was also found in pathways that, to our knowledge have not been associated previously with the key-pathophysiological signs of MPB such as melatonin signalling/degradation, adipogenesis and immune related pathways. Notably, both melatonin degradation and adipogenesis are reported to interact with, and to be controlled by, sex hormones^37^,^38^ and decreasing melatonin levels are a potential marker of puberty progression^39^. These findings highlight the indispensability of sex hormones to the MPB phenotype^40^, and point to a novel potential link between intrafollicular melatonin synthesis and its recognized effects on oestrogen receptor expression and thus the sensitivity of human scalp hair follicles to stimulation with oestrogens, which effectively counteract the anagen-shortening effects of DHT in MPB development^36^,^41^.

Regarding adipogenesis, maturation of adipocyte precursors in the skin is reported to occur in parallel with the activation of hair follicle stem cells and to drive anagen induction and hair growth during synchronized hair regeneration in mice^42^. However, few data are available on whether and in what manner adipogenesis in the skin may impact hair follicle cycling in humans. Notably, adipogenesis and follicular stem cell activation are impaired in mice lacking the gene *EBF1*, which is located at another MPB-risk locus on 5q33.3, which suggest that adipogenesis plays a role in healthy hair cycle regulation and MPB development in humans. Regarding immunological processes, no conclusive data on their role in MPB aetiology are yet available^43^. However, skin resident macrophages contribute to the cyclic activation of adult hair follicles via induction of WNT- and FGF5-signalling, which were also implicated in the present meta-analysis^44^,^45^, and perifollicular inflammatory infiltrates have long been suspected to participate in the terminal-to-vellus transformation in MBP^46^,^47^. Thus, our data represent the first genetic evidence that cells residing in the immediate hair follicle microenvironment impact on MPB development and may warrant investigation as therapeutic targets.

### Overlap with other human traits

On an epidemiological level, early-onset MPB has been associated with several severe late-onset somatic disorders, such as cardiovascular disease (CVD)^48^; prostate hyperplasia and cancer^49^,^50^,^51^; Parkinson's disease^10^; and amyotrophic lateral sclerosis^52^. We therefore investigated a possible genetic overlap between MPB and other phenotypes, and compared our data to reported GWAS signals from the NHGRI GWAS catalogue. The results are summarized in Supplementary Data 3. A total of 124 GWAS catalogue entries mapped to MPB-risk loci (*r*^2^≥0.3 and/or *D*′>0.8). These included the above mentioned associations with hormone-dependent traits and a reduced pigmentation of facial skin, scalp hair and eyes. As regards to the well-established associations between MPB and CVD and prostate cancer, a total of seven overlapping associations were identified. These were found between MPB and: (i) blood pressure (*N*=3); (ii) QT-interval length (*N*=1); (iii) atrial fibrillation (*N*=1); (iv) sudden cardiac arrest (*N*=1); (v) and prostate cancer (*N*=1). Here, our analysis confirmed the positive epidemiological association between prostate cancer and MPB at Xq12 (*AR/EDA2R*-locus), pointing towards a shared pathophysiological mechanism that may involve EDA2R-signalling and AR-transactivation^53^. Surprisingly, for the majority of CVD GWAS SNPs, the direction of effect for MPB and CVD differed, thus opposing the reported positive association between MPB and CVD at an epidemiological level. This was not the case for the positive associations between MPB and diastolic blood pressure at 4q21.21, and sudden cardiac arrest at 12q13.12. Here, further analyses are warranted to elucidate the exact underlying genes and biological pathways, and how they relate to the epidemiological findings. Notably, while the 4q21.21 locus pointed towards a positive association between MPB and diastolic blood pressure levels, opposite effect direction were observed for a second overlapping association between these traits on 5q33.3. This indicates that the effect direction of the genetic correlation between two complex traits may differ between individual loci or pathways. This finding highlights the need for systematic studies to assess not only the quantitative genetic overlap but also individual overlapping genetic factors and the underlying genes and pathways. In addition, associations with four loci were found for MPB and lower body height, which may be driven by an accelerated progression of puberty and premature induction of epiphyseal closure^54^. MPB-risk alleles at 17q21.31 and 6q22.32 were associated with increased bone mineral density, which may be a consequence of optimized UVR-induced vitamin D synthesis in subjects with MPB. This is consistent with the observation that MPB-associated alleles confer a reduced risk for immune related phenotypes, such as type 1 diabetes; multiple sclerosis (MS); and rheumatoid arthritis (Supplementary Data 3). An increased incidence of these diseases has been reported in subjects with poor vitamin D intake and low serum vitamin D levels^55^. Furthermore, a recent Mendelian-randomization study by Mokry *et al*.^56^, found an association between genetically lowered 25-hydroxyvitamin D levels and increased susceptibility to MS. Moreover, our data indicate shared genetic determinants for MPB and a reduced risk for ovarian cancer, colorectal cancer, and chronic lymphatic leukaemia, as well as overlapping associations with progressive supranuclear palsy and decreased intracranial volume. The indispensability of sex hormones for the MPB phenotype is supported by the identification of overlapping genetic association between MPB and other hormone-dependent traits, such as an earlier age-at-onset of menarche in females and earlier sexual maturation and higher serum androgen levels in males (6q22.32, 16p13.12, Xp22.31)^12^,^57^,^58^,^59^,^60^.

## Discussion

The present genome-wide meta-analysis identified 63 risk loci for MPB, and highlights highly plausible candidate genes that are likely to be implicated in the key-pathophysiological features of MPB, such as deregulation of anagen-to-catagen transition (*FGF5*, *EBF1*, *DKK2*, adipogenesis); increased androgen sensitivity (*SRD5A2*, melatonin signalling); and transformation of pigmented terminal hair into unpigmented vellus hair (*IRF4*). As demonstrated for *SRD5A2*, the GWAS approach is a valuable tool for drug target identification and the newly identified candidate genes and pathways, perhaps most notably *FGF5* and melatonin signalling are promising targets for the development of novel therapeutic options for MPB. In addition to confirming the involvement of well-established pathways that control hormonal status (androgen metabolism, oestrogen signalling) and hair follicle cycling (WNT-signalling, EGF-signalling), our data support the importance of less-well studied biological contexts, such as the involvement of perifollicular macrophages and adipocytes.

Moreover, our data provide molecular evidence that rather than being an isolated trait, MPB shares a substantial biological basis with numerous other human phenotypes. This may have major implications in terms of the evaluation of MPB as an early prognostic marker for different phenotypes such as prostate cancer, sudden cardiac arrest or neurodegenerative disorders, and for the repurposing of existing drugs for use in MPB therapy. The latter is illustrated by the fact that the efficacy of the two U.S. Food and Drug Administration (FDA) approved drugs, minoxidilTable 1 Table 2 and finasteride in MPB, was a serendipitous finding in patients administered these medications for hypertension and prostate hyperplasia. Finally, these novel insights into the genetic basis of MPB and its association with other traits may help to elucidate the evolutionary forces responsible for the relatively high prevalence of MPB in the European population.

## Methods

### Study participants

Participants were drawn from eight independent genome-wide association studies early-onset MPB samples: 23andMe (9,009 cases; 8,491 controls); Bonn (581 cases; 416 controls); CoLaus (622 cases; 655 controls); Nijmegen Biomedical Study (145 cases; 247 controls); QIMRB1 (216 cases; 1,162 controls); QIMRB2 (59 cases; 498 controls); THISEAS (52 cases; 150 controls); and TwinsUK (163 cases; 210 controls). A detailed description of the studies and their phenotype definitions is provided in Supplementary Note 1. All eight studies were approved by the respective institutional ethics review committees (specified in Supplementary Note 1), and written informed consent was obtained from all participants prior to inclusion.

### Genome-wide association analyses

A summary of the study specific genotyping platforms, imputation methods and GWAS is provided in Supplementary Table 1. For each of the eight studies fulfillment of the following two criteria was required: (i) a minor allele frequency (MAF) of **>**1%; and (ii) a call rate of >98%, a variance ratio of ≥0.3 (MACH) or a proper info statistic of ≥0.4 (IMPUTE2). Imputed data were analysed using logistic regression and the dosage data options of either PLINK or SNPTEST.

### Meta-analysis

The present meta-analysis was performed in accordance with the GWAS meta-analysis standards outlined in de Bakker *et al*.^61^ A fixed effects model was used to combine the logistic regression effect estimates of individual studies into a joint estimate, as implemented in METAL^62^. Results were cross-validated using the respective implementations in METAINTER and YAMAS. The Cochran's *Q* statistics and the *I*^2^ measurement were used to test for cross-study heterogeneity. *P*-values for the test of heterogeneity were calculated according to Higgins *et al*.^63^ The meta-analysis included a total of *N*=8,004,650 SNPs that were available in the 23andMe cohort and (i) at least four additional studies for non-X-chromosomal SNPs, and (ii) three additional studies for X-chromosomal SNPs. The QQ-plot of the meta-analysis and a plot of the across-study homogeneity are shown in Supplementary Figs 2 and 3.

### Identification of independent risk loci

SNP associations with a *P*-value of <10^−6^ (METAL) were extracted from the meta-analysis and filtered for a heterogeneity *P*-value (*P*_Het_) of <0.01. SNPs separated by a distances of ≤100 kb on the autosomes and ≤500 kb on the X-chromosome were assigned to the same genomic region. Loci were considered to be significantly associated with MPB if at least one SNP within the defined region showed an association to MPB at *P*<5 × 10^−8^ (METAL) and (i) had a MAF of ≥0.05; or (ii) was supported by at least one SNP in linkage disequilibrium (LD; *r*^2^>0.5), with a MAF of ≥0.05 and an association with MPB at *P*<10^−6^ (METAL). MPB-risk loci were considered to be independent if the lead SNPs of the regions showed an LD of *r*^2^<0.2.

### Test for polygenicity

For the meta-analysis overall, an inflation factor of *λ*=1.303 was observed. After removal of LD-SNPs surrounding the two major loci (chr20, chrX), the inflation decreased to *λ*=1.20. After exclusion of all 63 associated regions, the inflation decreased to *λ*=1.16. This indicates that a relevant proportion of the observed inflation is driven by genome-wide significant associations. Furthermore, it is reasonable to assume from this finding that the remaining inflation is driven primarily by true genetic association. This is consistent with previous reports of a polygenetic contribution to MPB, and with observations from meta-analyses of other complex genetic traits in which large numbers of common risk factors were identified^64^,^65^. To determine whether the inflation was attributable to polygenicity or to confounding factors, the LD score regression method^66^ was applied using LD Score v1.0.0. The analysis confirmed that most of the observed inflation is because of polygenicity (63.5%). The residual genomic inflation is *λ*_GC_=1.09, indicating that the observed association findings are not due to population stratification.

To estimate the proportion of potentially-false positive findings, association *P*-values (logistic regression) and effect directions for the 63 lead SNPs were compared between the 23andMe cohort (23andMe) and a meta-analysis of the remaining seven cohorts (MAwo23andMe) (Supplementary Table 8). For 62 of the 63 loci (98%) the analysis revealed a consistency of effect directions between 23andMe and MAwo23andMe. Of these loci, 76% (47/62) showed at least nominal significant association to MPB in the MAwo23andMe study (METAL), with 72% (34/47) also achieving significant association at *P*<0.05/63=7.9 × 10^−4^ (METAL *P*-value Bonferroni corrected for number of risk loci). Lastly, in contrast to the self-report based phenotyping used in the 23andMe cohort, phenotyping in the Bonn sample (BN) was based on clinical assessment. Thus for true positive association findings, stronger effect sizes would be expected on average in the BN sample. This was indeed the case: for 52 out of 61 lead SNPs available in the BN sample (85%) which would be highly unlikely if these loci included a relevant proportion of false positives. On the basis of these results, no correction was made for the residual inflation. The level of significance of the 63 MPB lead SNPs after double GC correction with the residual inflation factors from the LD score regression analyses is however indicated in Table 1.

### Definition of credible sets of SNPs

For each risk locus, a credible set of SNPs was defined, as reported in Ripke *et al*.^65^ (Supplementary Table 9). In brief, here the connection between the deviance and the single-variant test statistics is used to calculate the probability that a specific variant is associated with MPB for the given set of data. These probabilities are used to determine sets of variants within the risk loci—that is, credible sets of SNPs—which contain the causal variant 99% of the time.

### Estimation of heritability

The Bonn cohort was used to estimate the heritability of MPB, since individual genotypes were available. All imputed variants with an imputation info score of >0.6 were extracted, and the corresponding genotypes were called using a probability threshold of 0.9. The resulting dataset comprised 997 male individuals and 8,271,786 SNPs. Variants with a MAF of >0.01 and a genotype SNP-call rate of >95% were then filtered. Only SNPs with a *P*-value of >5% for the corresponding test of differences in missingness between cases and controls were retained. After outlier detection with EIGENSTRAT based on autosomal data and the exclusion of individuals based on a genetic similarity of >0.05, calculated by GCTA, we obtained the Genetic Relationship Matrix (GRM) for the autosomes and the X-chromosome with respect to 991 male individuals. With these two GRMs and 10 eigenvectors from the EIGENSTRAT step above, the heritability components were estimated using GCTA. The estimated heritability on the observed scale was 0.666 (0.234) for the autosomes and 0.277 (0.054) for the X-chromosome (standard errors in brackets). To obtain the estimated heritability on the liability scale, as described in Lee *et al*.^67^, a straightforward extension of this transformation was used to account for the extreme case and extreme control sampling. Table 2 in Hamilton^68^ was used to divide the population into four bins. On the basis of age structure and Hamilton-Norwood (HN) grade, the transformation was calculated under the assumption that controls were selected from the first bin and cases from the last, thus reflecting the extreme sampling scheme. The resulting factor was approximately 0.509. This generated an estimated heritability for autosomal chromosomes of 0.339 (0.119), and 0.141 (0.027) for the X-chromosome.

### Estimation of explained phenotypic variance

The amount of phenotypic variance explained by the 63 genome-wide significant risk loci was estimated as the correlation coefficient *r*^2^ in a multivariate linear model, in which the significant index SNPs were predictors and the outcome was modelled as 1=case, 0=control.

### Genotype-risk score analysis

Using the lead SNPs from the 63 genome-wide significant loci, a genotype-risk score was constructed based on the weighted number of susceptibility alleles in an independent replication sample from the Heinz–Nixdorff Recall (HNR) cohort (*N*=1,201). Individuals with HN grade>II (*N*=1,108) were defined as cases, and individuals with HN-grade I or II at age <65 years were defined as controls (*N*=93). The weights were established using the beta coefficient from the meta-analysis. The resulting genotype score was divided into four quartiles. The risk for MPB was then tested in each quartile using a logistic regression model and the lowest quartile as a reference. Two models were tested: (I) genotype-risk score; and (II) age-adjusted risk score. The results of this analysis are shown in (Table 2).

### DEPICT analysis

DEPICT was used to identify plausible candidate genes at each of the 63 risk loci. As recommended in Pers *et al*.^69^, all independent SNPs with a *P*-value of <5 × 10^−8^ were included in the DEPICT analysis. Independent SNP sets were generated by retaining the most significant SNP from each set of SNPs with a pairwise LD of *r*^2^>0.1 and a physical distance of <500 kb. Pairwise LD coefficients were computed based on the imputing panel used in the eight GWASs (1000 Genomes Project Phase I CEU, GBR and TSI genotype data). The results of these analyses are shown in Supplementary Tables 4–6.

### Identification of hair follicle eQTLs

DNA from peripheral blood and RNA from occipital scalp hair follicle samples were obtained from 125 volunteer healthy male donors of German descent (mean age 27.9 years). Genome-wide genotyping and imputation of blood DNA samples were performed using Illumina's Human OmniExpress-12v1.0 bead array and IMPUTE2 (1000 Genomes, Phase I, June 2014). Whole-transcriptome profiling was performed on Illumina's HT-12v4 bead arrays after amplification and biotinylation of the hair follicle-RNA using the TotalPrep-96 RNA Amplification Kit (Illumina, San Diego, CA, USA). Expression data were quantile normalized, and only probes with a detection *P*-value of <0.01 (Illumina GenomeStudio Software) in at least 5% of the samples were taken into account. The selected expression probes were subsequently filtered for: a unique alignment to the transcriptome; a perfect or good probe quality, as reported in the R package illuminaHumanv4.db; and mapping to an ENTREZ gene ID. After quality control and filtering, data for 14,687 expression probes and 6,593,881 SNPs were included in the eQTL analysis. Associations between gene expression levels and SNP genotypes in cis (distance between SNP and expression probe ≤1 Mb) was tested in MatrixEQTL using an additive linear regression model. Association tests were corrected for the top five principle components. All eQTL findings at a false discovery rate (FDR) of <0.001 were considered significant.

### mRNA and miRNA expression analysis

Whole-transcriptome profiling of hair follicle-RNA samples (*N*=125) was performed as described above. MiRNA profiling of hair follicle samples (*N*=25) was performed on the AffymetrixGeneChip miRNA 4.0 (Affymetrix, Santa Clara, CA, USA) using a total of 250 ng of hair follicle miRNA. Poly(A) tailing and biotinylation were performed with the AffymetrixGeneChip Hybridization, Wash and Stain Kit, in accordance with the manufacturer's instructions. Data were analysed using the Affymetrix Expression Console software (v.1.4) (Affymetrix). MiRNAs were defined as ‘present' (*N*=1,169) or ‘absent' (*N*=1,409) according to the implemented RMA (robust multichip average) and DABG (detected above background) methods.

### Identification of miRNA target genes at MPB-risk loci

MiRNA target genes at MPB-risk loci (±500 kb from MPB lead SNP) were identified using the miRWalk2.0 algorithm (last accessed 24 March 2016). Only validated target genes and genes that were predicted by the miRWalk algorithm and at least three additional implemented databases were taken into account.

### Overlap between eQTLs and MPB-risk variants

To identify regulatory effects at known and novel MPB-risk loci, the present meta-analysis data were compared with the eQTL data from: (i) the Blood eQTL Browser (http://genenetwork.nl/bloodeqtlbrowser; last accessed on 21 December 2015); (ii) the GTEx Browser (http://www.gtexportal.org/home); and (iii) an unpublished eQTL dataset from human hair follicle. MPB-risk variants were considered to coincide with an eQTL if the MPB lead SNP itself, or any SNP in *r*^2^>0.5, showed an eQTL effect. The complete list of overlapping eQTL findings is provided in Supplementary Table 2.

### Enhancer enrichment analysis

Enhancers (including promoters) were defined by chromatin immunoprecipitation-sequencing (ChIP-seq) peaks of histone 3 lysine 27 acetylation (H3K27ac). A total of 140 H3K27ac data sets were downloaded from the roadmap epigenomics project (http://www.roadmapepigenomics.org), and reprocessed using DFilter. Only peaks with *P*<10^−10^ (DFilter) were retained. Four in house data sets from a human balding and a non-balding dermal papilla cell line with and without 10 nM of DHT treatment were included^70^. In these lines, a H3K27ac antibody (Abcam, Cat. ab4729; 5 μg antibody per 100 μg chromatin) was used for ChIP. The MPB credible SNPs were overlapped with the enhancer (H3K27ac) sets of each of the 144 cell lineages and tissues. For tissue (or cell lineage) A, the remaining 143 enhancer sets were pooled together to define a tissue agnostic superset. The superset was classified in feature categories based on: (i) distance to transcription start site; (ii) GC content; (iii) length of the enhancer site. To obtain for the enhancer set of tissue A an appropriate null distribution, we randomly drew for every enhancer of tissue A an enhancer from a matched feature category of the superset. The number of credible SNP overlaps across this matched enhancer set was computed, and a million permutations of feature matched enhancer sets were conducted and overlapped with the credible SNP set to define a distribution for tissue A. This procedure was conducted for each of the 144 tissue specific enhancer sets. For Fig. 2, enhancers of cell lineages/tissues that belonged to the same tissue type were merged, resulting in 23 grouped enhancer sets. The above framework was repeated for these grouped enhancer sets. The results of these analyses are provided in Supplementary Data 1.

### Ingenuity pathway enrichment analysis

To test for an enrichment of MPB candidate genes in canonical pathways, the Ingenuity Pathway Analysis was used (Qiagen, Hilden, Germany). IPA considers 658 pathways, and calculates enrichment based on the right-tailed Fisher's exact test. All genes within 500 kb of the MPB lead SNPs were included in the analysis. Only pathways with ≥3 annotated genes were taken into account. The complete list of nominally significant pathways is provided in Supplementary Table 7.

### NHGRI GWAS catalogue

Previously reported GWAS associations that mapped to the associated regions and showed an *r*^2^≥0.3 and/or *D*′>0.8 (1000 Genomes Project, Phase 3) with the respective MPB index SNP were extracted from the NHGRI GWAS catalogue (http://www.ebi.ac.uk/gwas/; last accessed on 14 January 2016). This resulted in the identification of 124 overlapping associations, as shown in Supplementary Data 3.

### Data availability

All data that support the findings of this study are available from the corresponding author upon reasonable request.

## Additional information

**How to cite this article:** Heilmann-Heimbach, S. *et al*. Meta-analysis identifies novel risk loci and yields systematic insights into the biology of male-pattern baldness. *Nat. Commun.* **8,** 14694 doi: 10.1038/ncomms14694 (2017).

**Publisher's note:** Springer Nature remains neutral with regard to jurisdictional claims in published maps and institutional affiliations.

## Supplementary Material

Supplementary Information Supplementary Figures, Supplementary Tables, Supplementary Note, and Supplementary References

Supplementary Data 1 Results of the enhancer enrichment analysis of MPB credible SNPs at the 63 genome-wide significant risk loci.

Supplementary Data 2 Functional description of genes that were identified as plausible candidate genes based on at least two of the following criteria: expression in human hair follicle (E), functional evidence from literature (F), vicinity to MPB lead SNP (N), evidence for cis-eQTL effect (Q), evidence from DEPICT analysis (D) (see Table 1). Chr. – Chromosome; PMID – relevant PubMed-IDs

Supplementary Data 3 Shared genetic determinants between MPB and other human traits. MPB SNP - MPB lead SNP; GWAS SNP - reported GWAS SNP; CHR - chromosome, BP - base pair; EA - effect allele; OR - odds ratio; BETA - effect size; CI - confidence interval; MPB EA GWAS SNP - effect allele of GWAS SNP in MPB meta-analysis; LD - linkage desequilibrium; NR- not reported; + - MPB risk increasing allele increases risk for reported phenotype; - - MPB risk increasing allele decreases risk for reported phenotype

## Figures and Tables

**Table 1:**
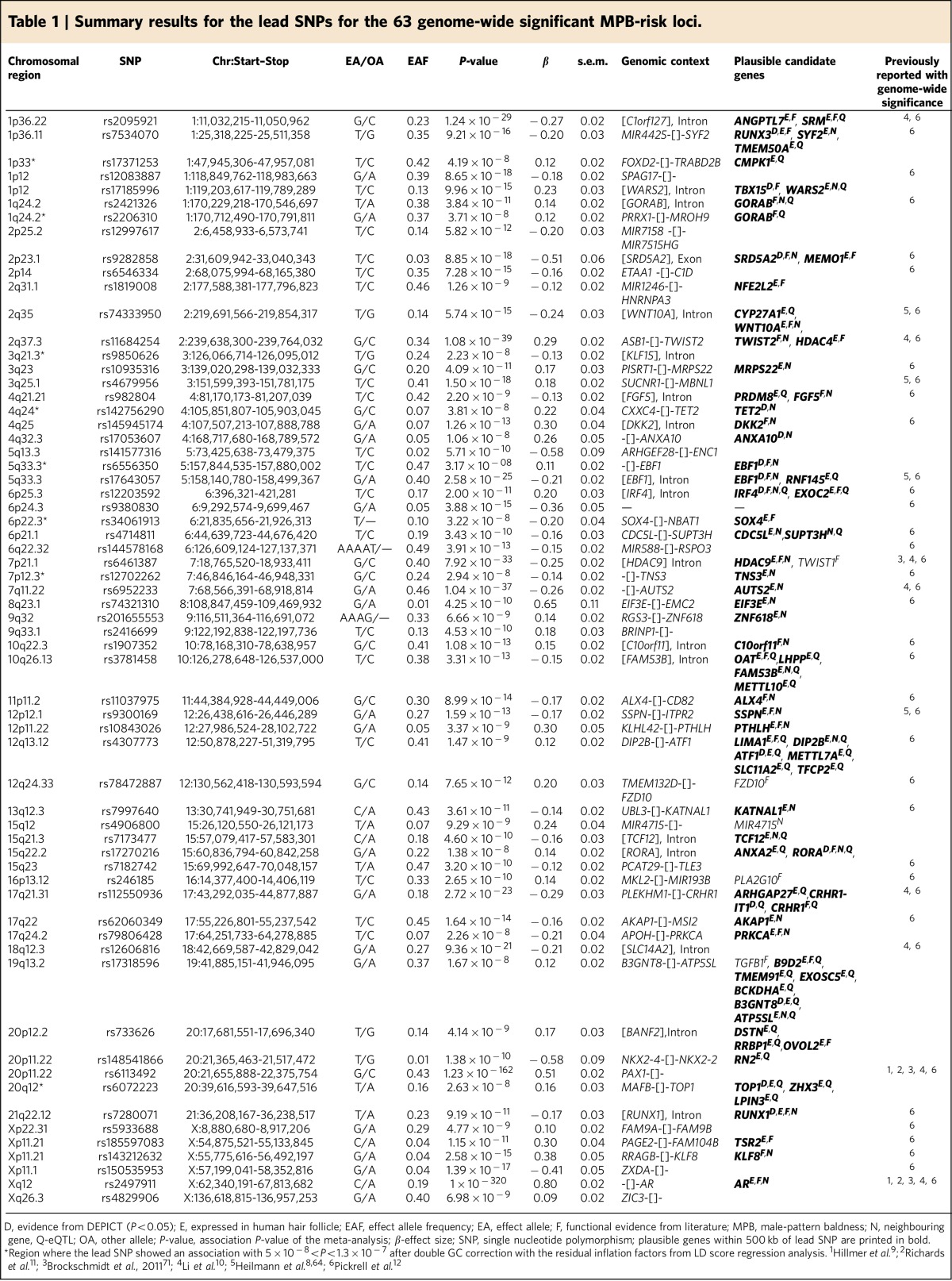
Summary results for the lead SNPs for the 63 genome-wide significant MPB-risk loci.

*Reaction conditions:**1**/LiHMDS/**2**/[Pd(*η*^3^-C_3_H_5_)Cl]_2_/S-IPr·HCl=200/200/100/2.5/5; 0.1 M of ketone **1**; T=30^o^C; B/L and *dr* was determined by ^1^H NMR, *dr* is the ratio of (±)-(*syn,anti*)-**3**/other diastereoisomers; Isolated yield. †T=50 ^o^C. ‡Solvent=THF. §OBoc of **2** was replaced with OP(OEt)_2_. ||The yield was determined by ^1^H NMR.

**Table 2:** Association between the weighted genotype-risk score and MPB.

	**Model I**		**Model II**	
	**OR**	**95% CI**	**OR**	**95% CI**
Quartile 1	1.00 (reference)		1.00 (reference)	
Quartile 2	1.547	0.90–2.65	1.87	1.06–3.32
Quartile 3	1.796	1.03–3.13	2.12	1.17–3.83
Quartile 4	4.16	2.03–8.55	5.14	2.45–10.86

95% CI, 95% confidence interval; Model I, weighted genotype-risk score; Model II, age-adjusted weighted genotype-risk score; MPB, male-pattern baldness; OR, odds ratio.

## References

[b1] Qi J. & Garza L. A. An overview of alopecias. Cold Spring Harb. Perspect. Med. 4, a013615 (2014). 24591533 10.1101/cshperspect.a013615 PMC3935391

[b2] Han J. . A genome-wide association study identifies novel alleles associated with hair color and skin pigmentation. PLoS Genet. 4, e1000074 (2008). 18483556 10.1371/journal.pgen.1000074 PMC2367449

[b3] Yu N. L., Tan H., Song Z. Q. & Yang X. C. Illness perception in patients with androgenetic alopecia and alopecia areata in China. J. Psychosom. Res. 86, 1–6 (2016). 27302539 10.1016/j.jpsychores.2016.04.005

[b4] Heilmann-Heimbach S., Hochfeld L. M., Paus R. & Nothen M. M. Hunting the genes in male-pattern alopecia: how important are they, how close are we and what will they tell us? Exp. Dermatol. 25, 251–257 (2016). 26843402 10.1111/exd.12965

[b5] Inui S. & Itami S. Androgen actions on the human hair follicle: perspectives. Exp. Dermatol. 22, 168–171 (2013). 23016593 10.1111/exd.12024

[b6] Ganzer C. A., Jacobs A. R. & Iqbal F. Persistent sexual, emotional, and cognitive impairment post-finasteride: a survey of men reporting symptoms. Am. J. Mens Health 9, 222–228 (2015). 24928450 10.1177/1557988314538445

[b7] Plenge R. M., Scolnick E. M. & Altshuler D. Validating therapeutic targets through human genetics. Nat. Rev. Drug. Discov. 12, 581–594 (2013). 23868113 10.1038/nrd4051

[b8] Heilmann S. . Androgenetic alopecia: identification of four genetic risk loci and evidence for the contribution of WNT signaling to its etiology. J. Invest. Dermatol. 133, 1489–1496 (2013). 23358095 10.1038/jid.2013.43

[b9] Hillmer A. M. . Susceptibility variants for male-pattern baldness on chromosome 20p11. Nat. Genet. 40, 1279–1281 (2008). 18849994 10.1038/ng.228

[b10] Li R. . Six novel susceptibility loci for early-onset androgenetic alopecia and their unexpected association with common diseases. PLoS Genet. 8, e1002746 (2012). 22693459 10.1371/journal.pgen.1002746 PMC3364959

[b11] Richards J. B. . Male-pattern baldness susceptibility locus at 20p11. Nat. Genet. 40, 1282–1284 (2008). 18849991 10.1038/ng.255 PMC2672151

[b12] Pickrell J. K. . Detection and interpretation of shared genetic influences on 42 human traits. Nat. Genet. 48, 1296 (2016). 10.1038/ng1016-1296a 27681293

[b13] Nyholt D. R., Gillespie N. A., Heath A. C. & Martin N. G. Genetic basis of male pattern baldness. J. Invest. Dermatol. 121, 1561–1564 (2003). 14675213 10.1111/j.1523-1747.2003.12615.x

[b14] Rexbye H. . Hair loss among elderly men: etiology and impact on perceived age. J. Gerontol. A Biol. Sci. Med. Sci. 60, 1077–1082 (2005). 16127116 10.1093/gerona/60.8.1077

[b15] Consortium GTEx. Human genomics. The genotype-tissue expression (GTEx) pilot analysis: multitissue gene regulation in humans. Science 348, 648–660 (2015). 25954001 10.1126/science.1262110 PMC4547484

[b16] Westra H. J. . Systematic identification of trans eQTLs as putative drivers of known disease associations. Nat. Genet. 45, 1238–1243 (2013). 24013639 10.1038/ng.2756 PMC3991562

[b17] Paus R., Langan E. A., Vidali S., Ramot Y. & Andersen B. Neuroendocrinology of the hair follicle: principles and clinical perspectives. Trends Mol. Med. 20, 559–570 (2014). 25066729 10.1016/j.molmed.2014.06.002

[b18] Kizil C. . Simplet/Fam53b is required for Wnt signal transduction by regulating beta-catenin nuclear localization. Development 141, 3529–3539 (2014). 25183871 10.1242/dev.108415

[b19] Andl T. & Botchkareva N. V. MicroRNAs (miRNAs) in the control of HF development and cycling: the next frontiers in hair research. Exp. Dermatol. 24, 821–826 (2015). 26121602 10.1111/exd.12785 PMC5721351

[b20] Hebert J. M., Rosenquist T., Gotz J. & Martin G. R. FGF5 as a regulator of the hair growth cycle: evidence from targeted and spontaneous mutations. Cell 78, 1017–1025 (1994). 7923352 10.1016/0092-8674(94)90276-3

[b21] Higgins C. A. . FGF5 is a crucial regulator of hair length in humans. Proc. Natl Acad. Sci. USA 111, 10648–10653 (2014). 24989505 10.1073/pnas.1402862111 PMC4115575

[b22] Ota Y. . Fibroblast growth factor 5 inhibits hair growth by blocking dermal papilla cell activation. Biochem. Biophys. Res. Commun. 290, 169–176 (2002). 11779149 10.1006/bbrc.2001.6140

[b23] Kwack M. H., Kim M. K., Kim J. C. & Sung Y. K. Dickkopf 1 promotes regression of hair follicles. J. Invest. Dermatol. 132, 1554–1560 (2012). 22358062 10.1038/jid.2012.24

[b24] Kwack M. H. . Dihydrotestosterone-inducible dickkopf 1 from balding dermal papilla cells causes apoptosis in follicular keratinocytes. J. Invest. Dermatol. 128, 262–269 (2008). 17657240 10.1038/sj.jid.5700999

[b25] Inui S. & Itami S. Molecular basis of androgenetic alopecia: From androgen to paracrine mediators through dermal papilla. J. Dermatol. Sci. 61, 1–6 (2011). 21167691 10.1016/j.jdermsci.2010.10.015

[b26] Eriksson N. . Web-based, participant-driven studies yield novel genetic associations for common traits. PLoS Genet. 6, e1000993 (2010). 20585627 10.1371/journal.pgen.1000993 PMC2891811

[b27] Sulem P. . Two newly identified genetic determinants of pigmentation in Europeans. Nat. Genet. 40, 835–837 (2008). 18488028 10.1038/ng.160

[b28] Praetorius C. . A polymorphism in IRF4 affects human pigmentation through a tyrosinase-dependent MITF/TFAP2A pathway. Cell 155, 1022–1033 (2013). 24267888 10.1016/j.cell.2013.10.022 PMC3873608

[b29] Sturm R. A. Molecular genetics of human pigmentation diversity. Hum. Mol. Genet. 18, R9–R17 (2009). 19297406 10.1093/hmg/ddp003

[b30] Stough D. . Psychological effect, pathophysiology, and management of androgenetic alopecia in men. Mayo Clin. Proc. 80, 1316–1322 (2005). 16212145 10.4065/80.10.1316

[b31] Jain R. & De-Eknamkul W. Potential targets in the discovery of new hair growth promoters for androgenic alopecia. Expert Opin. Ther. Targets 18, 787–806 (2014). 24873677 10.1517/14728222.2014.922956

[b32] Schweikert H. U. & Wilson J. D. Regulation of human hair growth by steroid hormones. I. Testerone metabolism in isolated hairs. J. Clin. Endocrinol. Metab. 38, 811–819 (1974). 4823922 10.1210/jcem-38-5-811

[b33] Hillmer A. M. . Genetic variation in the human androgen receptor gene is the major determinant of common early-onset androgenetic alopecia. Am. J. Hum. Genet. 77, 140–148 (2005). 15902657 10.1086/431425 PMC1226186

[b34] Wehner G. & Schweikert H. U. Estrone sulfate source of estrone and estradiol formation in isolated human hair roots: identification of a pathway linked to hair growth phase and subject to site-, gender-, and age-related modulations. J. Clin. Endocrinol. Metab. 99, 1393–1399 (2014). 24432990 10.1210/jc.2013-2607

[b35] Mak K. K. & Chan S. Y. Epidermal growth factor as a biologic switch in hair growth cycle. J. Biol. Chem. 278, 26120–26126 (2003). 12714603 10.1074/jbc.M212082200

[b36] Ohnemus U., Uenalan M., Inzunza J., Gustafsson J. A. & Paus R. The hair follicle as an estrogen target and source. Endocr. Rev. 27, 677–706 (2006). 16877675 10.1210/er.2006-0020

[b37] Li C. & Zhou X. Melatonin and male reproduction. Clin. Chim. Acta 446, 175–180 (2015). 25916694 10.1016/j.cca.2015.04.029

[b38] Zerradi M., Dereumetz J., Boulet M. M. & Tchernof A. Androgens, body fat distribution and adipogenesis. Curr. Obes. Rep. 3, 396–403 (2014). 26626916 10.1007/s13679-014-0119-6

[b39] Crowley S. J., Acebo C. & Carskadon M. A. Human puberty: salivary melatonin profiles in constant conditions. Dev. Psychobiol. 54, 468–473 (2012). 21953482 10.1002/dev.20605 PMC4167613

[b40] Hamilton J. B. Effect of castration in adolescent and young adult males upon further changes in the proportions of bare and hairy scalp. J. Clin. Endocrinol. Metab. 20, 1309–1318 (1960). 13711016 10.1210/jcem-20-10-1309

[b41] Kobayashi H. . A role of melatonin in neuroectodermal-mesodermal interactions: the hair follicle synthesizes melatonin and expresses functional melatonin receptors. FASEB J. 19, 1710–1712 (2005). 16030176 10.1096/fj.04-2293fje

[b42] Festa E. . Adipocyte lineage cells contribute to the skin stem cell niche to drive hair cycling. Cell 146, 761–771 (2011). 21884937 10.1016/j.cell.2011.07.019 PMC3298746

[b43] Trueb R. M. Molecular mechanisms of androgenetic alopecia. Exp. Gerontol. 37, 981–990 (2002). 12213548 10.1016/s0531-5565(02)00093-1

[b44] Castellana D., Paus R. & Perez-Moreno M. Macrophages contribute to the cyclic activation of adult hair follicle stem cells. PLoS Biol. 12, e1002002 (2014). 25536657 10.1371/journal.pbio.1002002 PMC4275176

[b45] Suzuki S. . Localization of rat FGF-5 protein in skin macrophage-like cells and FGF-5S protein in hair follicle: possible involvement of two Fgf-5 gene products in hair growth cycle regulation. J. Invest. Dermatol. 111, 963–972 (1998). 9856803 10.1046/j.1523-1747.1998.00427.x

[b46] Jaworsky C., Kligman A. M. & Murphy G. F. Characterization of inflammatory infiltrates in male pattern alopecia: implications for pathogenesis. Br. J. Dermatol. 127, 239–246 (1992). 1390168 10.1111/j.1365-2133.1992.tb00121.x

[b47] Piérard G., Piérard-Franchimont C., Nikkels-Tassoudji N., Nikkels A. & Léger D. S. Improvement in the inflammatory aspect of androgenetic alopecia. A pilot study with an antimicrobial lotion. J. Dermatol. Treat. 7, 153–157 (1996).

[b48] Trieu N. & Eslick G. D. Alopecia and its association with coronary heart disease and cardiovascular risk factors: a meta-analysis. Int. J. Cardiol. 176, 687–695 (2014). 25150481 10.1016/j.ijcard.2014.07.079

[b49] Arias-Santiago S. . Androgenetic alopecia as an early marker of benign prostatic hyperplasia. J. Am. Acad. Dermatol. 66, 401–408 (2012). 21835498 10.1016/j.jaad.2010.12.023

[b50] Hawk E., Breslow R. A. & Graubard B. I. Male pattern baldness and clinical prostate cancer in the epidemiologic follow-up of the first National Health and Nutrition Examination Survey. Cancer Epidemiol. Biomarkers Prev. 9, 523–527 (2000). 10815699

[b51] Amoretti A., Laydner H. & Bergfeld W. Androgenetic alopecia and risk of prostate cancer: A systematic review and meta-analysis. J. Am. Acad. Dermatol. 68, 937–943 (2013). 23395589 10.1016/j.jaad.2012.11.034

[b52] Fondell E., Fitzgerald K. C., Falcone G. J., O'Reilly E. J. & Ascherio A. Early-onset alopecia and amyotrophic lateral sclerosis: a cohort study. Am. J. Epidemiol. 178, 1146–1149 (2013). 23942216 10.1093/aje/kwt096 PMC3783095

[b53] Ryan C. J. & Chan J. M. Hair, hormones, and high-risk prostate cancer. J. Clin. Oncol. 33, 386–387 (2015). 25547509 10.1200/JCO.2014.58.5588

[b54] Vanderschueren D., Gaytant J., Boonen S. & Venken K. Androgens and bone. Curr. Opin. Endocrinol. Diabetes Obes. 15, 250–254 (2008). 18438173 10.1097/MED.0b013e3282fe6ca9

[b55] Khan Q. J. & Fabian C. J. How I treat vitamin D deficiency. J. Oncol. Pract. 6, 97–101 (2010). 20592785 10.1200/JOP.091087 PMC2835491

[b56] Mokry L. E. . Vitamin D and risk of multiple sclerosis: a mendelian randomization study. PLoS Med. 12, e1001866 (2015). 26305103 10.1371/journal.pmed.1001866 PMC4549308

[b57] Cousminer D. L. . Genome-wide association study of sexual maturation in males and females highlights a role for body mass and menarche loci in male puberty. Hum. Mol. Genet. 23, 4452–4464 (2014). 24770850 10.1093/hmg/ddu150 PMC4168307

[b58] Elks C. E. . Thirty new loci for age at menarche identified by a meta-analysis of genome-wide association studies. Nat. Genet. 42, 1077–1085 (2010). 21102462 10.1038/ng.714 PMC3140055

[b59] Jin G. . Genome-wide association study identifies a new locus JMJD1C at 10q21 that may influence serum androgen levels in men. Hum. Mol. Genet. 21, 5222–5228 (2012). 22936694 10.1093/hmg/dds361 PMC3607470

[b60] Perry J. R. . Parent-of-origin-specific allelic associations among 106 genomic loci for age at menarche. Nature 514, 92–97 (2014). 25231870 10.1038/nature13545 PMC4185210

[b61] de Bakker P. I. W. . Practical aspects of imputation-driven meta-analysis of genome-wide association studies. Hum. Mol. Genet. 17, R122–R128 (2008). 18852200 10.1093/hmg/ddn288 PMC2782358

[b62] Willer C. J., Li Y. & Abecasis G. R. METAL: fast and efficient meta-analysis of genomewide association scans. Bioinformatics 26, 2190–2191 (2010). 20616382 10.1093/bioinformatics/btq340 PMC2922887

[b63] Higgins J. P., Thompson S. G., Deeks J. J. & Altman D. G. Measuring inconsistency in meta-analyses. BMJ 327, 557–560 (2003). 12958120 10.1136/bmj.327.7414.557 PMC192859

[b64] Heilmann S. . Evidence for a polygenic contribution to androgenetic alopecia. Br. J. Dermatol. 169, 927–930 (2013). 23701444 10.1111/bjd.12443

[b65] Ripke S. . Biological insights from 108 schizophrenia-associated genetic loci. Nature 511, 421–427 (2014). 25056061 10.1038/nature13595 PMC4112379

[b66] Bulik-Sullivan B. . An atlas of genetic correlations across human diseases and traits. Nat. Genet. 47, 1236–1241 (2015). 26414676 10.1038/ng.3406 PMC4797329

[b67] Lee S. H., Wray N. R., Goddard M. E. & Visscher P. M. Estimating missing heritability for disease from genome-wide association studies. Am. J. Hum. Genet. 88, 294–305 (2011). 21376301 10.1016/j.ajhg.2011.02.002 PMC3059431

[b68] Hamilton J. B. Patterned loss of hair in man; types and incidence. Ann. N.Y. Acad. Sci. 53, 708–728 (1951). 14819896 10.1111/j.1749-6632.1951.tb31971.x

[b69] Pers T. H. . Biological interpretation of genome-wide association studies using predicted gene functions. Nat. Commun. 6, 5890 (2015). 25597830 10.1038/ncomms6890 PMC4420238

[b70] Chew E. G. . Differential expression between human dermal papilla cells from balding and non-balding scalps reveals new candidate genes for androgenetic alopecia. J. Invest. Dermatol. 136, 1559–1567 (2016). 27060448 10.1016/j.jid.2016.03.032

[b71] Brockschmidt F. F. . Susceptibility variants on chromosome 7p21.1 suggest HDAC9 as a new candidate gene for male-pattern baldness. Br. J. Dermatol. 165, 1293–1302 (2011). 22032556 10.1111/j.1365-2133.2011.10708.x

