# Supplementary material for: Meta-analysis identifies novel risk loci and yields systematic insights into the biology of male-pattern baldness
Source: Nat Commun. 2017 Mar 8;8:14694. doi: 10.1038/ncomms14694 (PMC5344973; doi:10.1038/ncomms14694)
Supplement: Supplementary Information — Supplementary Figures, Supplementary Tables, Supplementary Note, and Supplementary References [file ncomms14694-s1.pdf]

Supplementary Note 1

Descriptions of the eight included studies and respective phenotype definitions

Bonn: The Bonn male pattern baldness (MPB) cohort comprised 581 cases and 416 controls. All subjects were of Central European descent. Men were considered to have early-onset MPB if they had developed Hamilton-Norwood (HN) grade IV-VII hair loss at ≤30 years or HN V-VII at <40 years of age. The control samples comprised: (i) 270 men aged ≥60 years without hair loss (HN I, unaffected controls); and (ii) 146 male controls from a population-based study (45-63 years, HN I-V). The 146 population-based controls were recruited from the Heinz Nixdorf Recall cohort at the University of Essen[1,2]. All cases and the remaining 270 unaffected controls were recruited by dermatologists at the Department of Dermatology, University of Düsseldorf. The study was approved by the Ethics Committees of the Universities of Bonn, Düsseldorf and Essen, and informed consent was obtained from all participating individuals.

CoLaus: Subjects for the CoLaus cohort were selected at random from the permanent residents of Lausanne, Switzerland between 2003 and 2006[3,4]. Men were considered to have MPB if they had developed HN≥V between the ages of 35 and 65 years. Control status was defined as HN I between ages 45 and 55 years or HN II between the ages of 56 and 75 years. All participants were recruited and assessed by trained nurses. Age of onset was assessed by questionnaire. A total of 622 early onset MPB cases and 655 controls from the CoLaus cohort were included in the present study. The study was approved by the faculty of medicine Ethics Committee of the University of Lausanne and informed consent was obtained from all participating individuals.

TwinsUK: The TwinsUK cohort is a population-based sample of Britons, unselected for any disease or trait, which is representative of the UK singleton population[5].All male subjects were assessed for MPB by a dermatologist using standardized photographs. Case status was defined as HN V-VII hair loss. Control status was defined as HN I. A total of 163 early onset MPB cases and 210 controls[6] from the TwinsUK cohort were included in the present study. The study was approved by St. Thomas' Hospital Research Ethics Committee (EC96/439

Twins UK) and all participants provided written informed consent.

Nijmegen Biomedical Study: Details of this population-based study of gene-environment interactions in multifactorial disease are provided elsewhere[7]. The cohort comprised 9,371 individuals drawn from 22,500 age and sex stratified, randomly selected residents of Nijmegen. All participants were of self-reported European descent. Male participants were asked to match their hair pattern at the ages of 20 years and 40 years, as well as age at study entry, to a HN grading schema[8]. MPB was defined as the presence of HN IV-VII by age 40 years. Control status was defined as HN I at age ≥50 years, or HN II-III at age ≥60 years. A total of 145 early onset MPB cases and 247 controls from the Nijmegen Biomedical Study cohort were included in the present study. The study was approved by the Institutional Review Board of the Radboud University Medical Center (RUNMC) and informed consent was obtained from all participating individuals.

23andMe: Participants from this cohort are unrelated male individuals of predominantly European ancestry, selected from consented research participants of 23andMe Inc.[9] The androgenetic alopecia status was measured by self-evaluation through a web-based survey. Cases were defined as individuals with Hamilton grade ≥III and age of onset < 40, while individuals with Hamilton grade I and age ≥ 30, or Hamilton grade II and age ≥50 were considered as controls. In the current study 9,009 cases and 8,491 controls were included[10]. The study was approved by Ethical & Independent Review Services, an AAHRPP accredited institutional review board and all participants provided informed consent.

Australian population based twin study: Measures of hair loss were obtained in the course of an extensive semi-structured telephone interview with respondent booklet, designed to assess physical, psychological and social manifestations of alcoholism and related disorders, conducted with 6265 twins born 1964-71 from the volunteer based Australian Twin Registry. All males (45% of the sample) were asked to rate their degree of hair loss, if any, according to the HN scale, which was printed in the respondent booklet. This data collection scheme was validated in a previous study[11]. Individuals with hair loss Hamilton-Norwood type III or greater were classified as cases and population based individuals were recruited as controls. For the purposes of the present study, two early onset MPB cohorts were used. The first cohort (QIMRB1) comprised 216 unrelated cases and 1,162 unrelated controls (HumanHap generation arrays). The second cohort (QIMRB2) comprised 59 unrelated cases and 498

controls (Omni generation arrays). The study was approved by the Human Research Ethics Committee at QIMR Berghofer Medical Research Institute and written informed consent was obtained from all participants.

THISEAS: The Hellenic Study of Interactions between SNPs and Eating in Atherosclerosis Susceptibility (THISEAS) is a medical center based case- control study of coronary artery disease in Greek adults. Hair loss status of male participants was assessed by trained scientists using the HN scale. Early onset MPB was defined as HN≥III at age<50 years. Control status was defined as a maximum of HN II at age ≥50 years. A total of 150 early onset MPB cases and 52 controls from the THISEAS cohort were included in the present study[12]. The study was approved by the Ethics Committee of the Harokopio University and all participants gave their written informed consent.

a

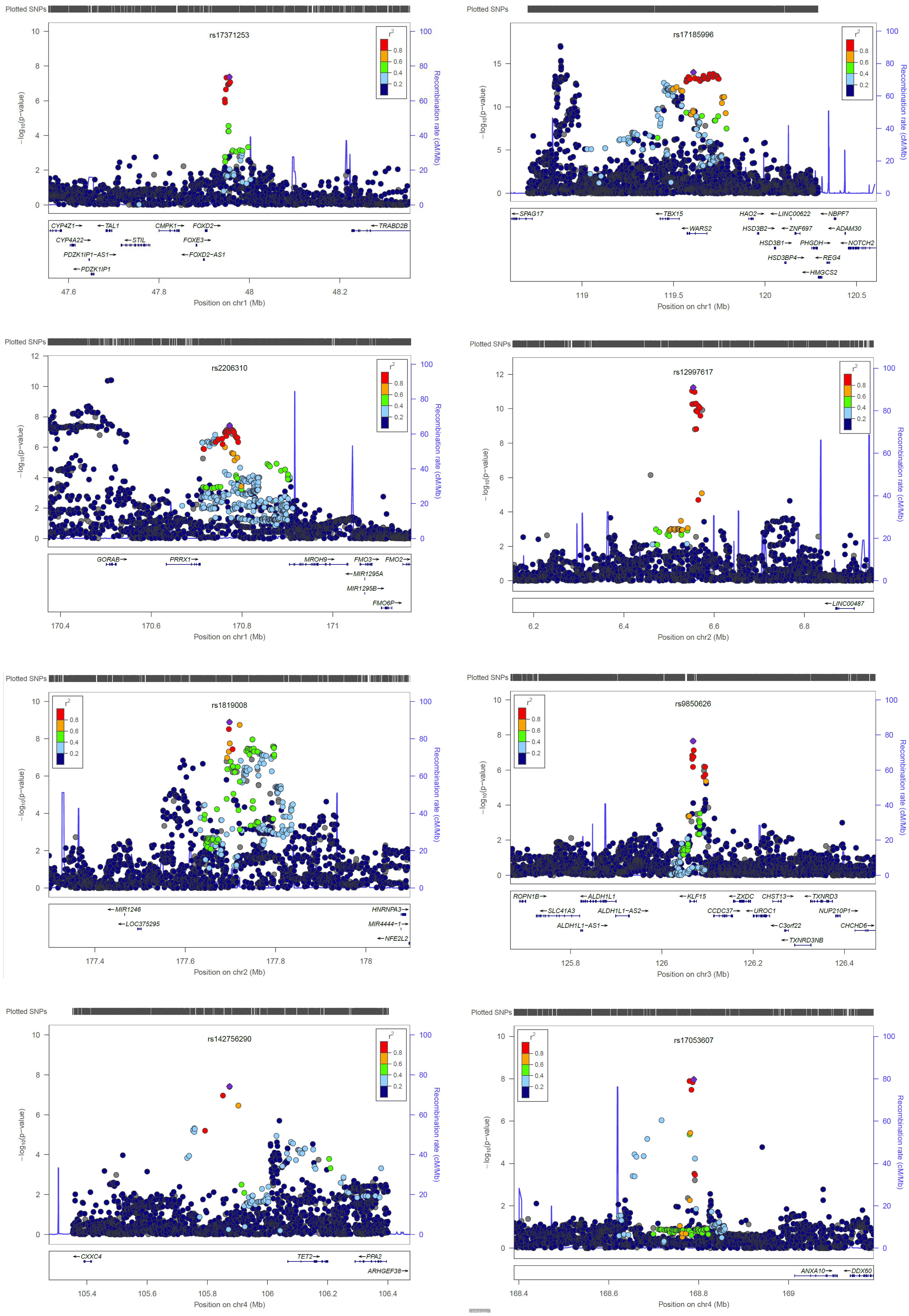

Plotted SNPs
rs17371253
−log10(p−value)
Recombination rate (cM/Mb)
CYP4Z1
TAL1
CMPK1
FOXD2
TRABD2B
CYP4A22
STIL
FOXE3
PDZK1IP1−AS1
FOXD2−AS1
PDZK1IP1
Position on chr1 (Mb)
rs17185996
SPAG17
TBX15
WARS2
HAO2
LINC00622
NBPF7
HSD3B2
ZNF697
ADAM30
HSD3B1
PHGDH
NOTCH2
HSD3BP4
REG4
HMGCS2
Position on chr1 (Mb)
rs2206310
GORAB
PRRX1
MROH9
FMO3
FMO2
MIR1295A
MIR1295B
FMO6P
Position on chr1 (Mb)
rs12997617
LINC00487
Position on chr2 (Mb)
rs1819008
MIR1246
LOC375295
HNRNPA3
MIR4444−1
NFE2L2
Position on chr2 (Mb)
rs9850626
ROPN1B
ALDH1L1
KLF15
ZXDC
CHST13
TXNRD3
SLC41A3
ALDH1L1−AS2
CCDC37
UROC1
NUP210P1
ALDH1L1−AS1
C3orf22
CHCHD6
TXNRD3NB
Position on chr3 (Mb)
rs142756290
CXXC4
TET2
PPA2
ARHGEF38
Position on chr4 (Mb)
rs17053607
ANXA10
DDX60
Position on chr4 (Mb)

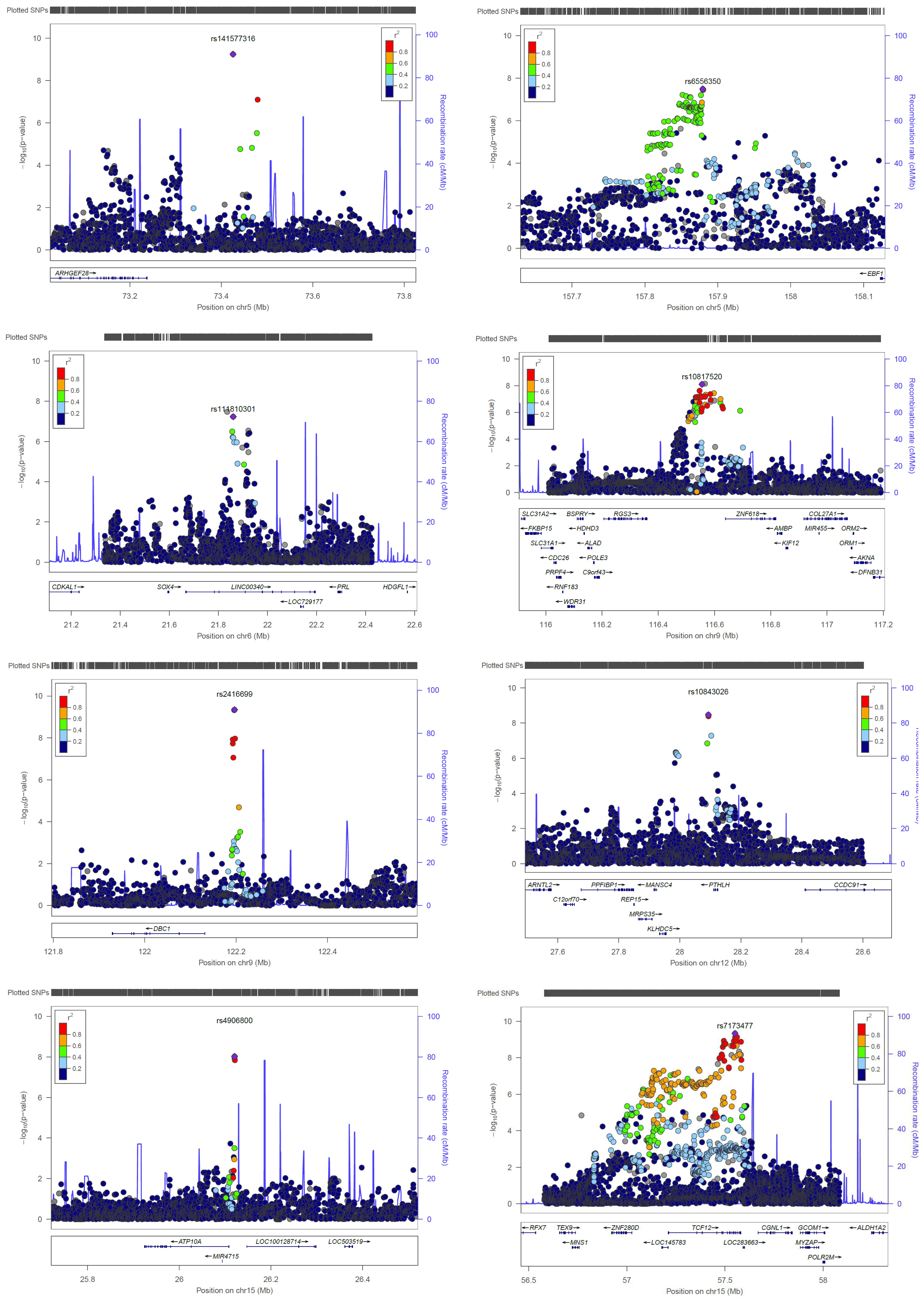

Plotted SNPs
rs141577316
−log10(p−value)
Recombination rate (cM/Mb)
ARHGEF28→
73.2
73.4
73.6
73.8
Position on chr5 (Mb)
rs6556350
←EBF1
157.7
157.8
157.9
158
158.1
Position on chr5 (Mb)
rs111810301
CDKAL1→
SOX4→
LINC00340→
←PRL
HDGFL1→
←LOC729177
21.2
21.4
21.6
21.8
22
22.2
22.4
22.6
Position on chr6 (Mb)
rs10817520
SLC31A2→
BSPRY→
RGS3→
ZNF618→
COL27A1→
←FKBP15
←HDHD3
←AMBP
MIR455→
ORM2→
SLC31A1→
←ALAD
←KIF12
ORM1→
←CDC26
←POLE3
←AKNA
PRPF4→
C9orf43→
←DFNB31
←RNF183
←WDR31
116
116.2
116.4
116.6
116.8
117
117.2
Position on chr9 (Mb)
rs2416699
←DBC1
121.8
122
122.2
122.4
Position on chr9 (Mb)
rs10843026
ARNTL2→
PPFIBP1→
←MANSC4
←PTHLH
CCDC91→
C12orf70→
REP15→
MRPS35→
KLHDC5→
27.6
27.8
28
28.2
28.4
28.6
Position on chr12 (Mb)
rs4906800
←ATP10A
LOC100128714→
LOC503519→
←MIR4715
25.8
26
26.2
26.4
Position on chr15 (Mb)
rs7173477
←RFX7
TEX9→
←ZNF280D
TCF12→
CGNL1→
GCOM1→
←ALDH1A2
←MNS1
←LOC145783
LOC283663→
MYZAP→
POLR2M→
56.5
57
57.5
58
Position on chr15 (Mb)

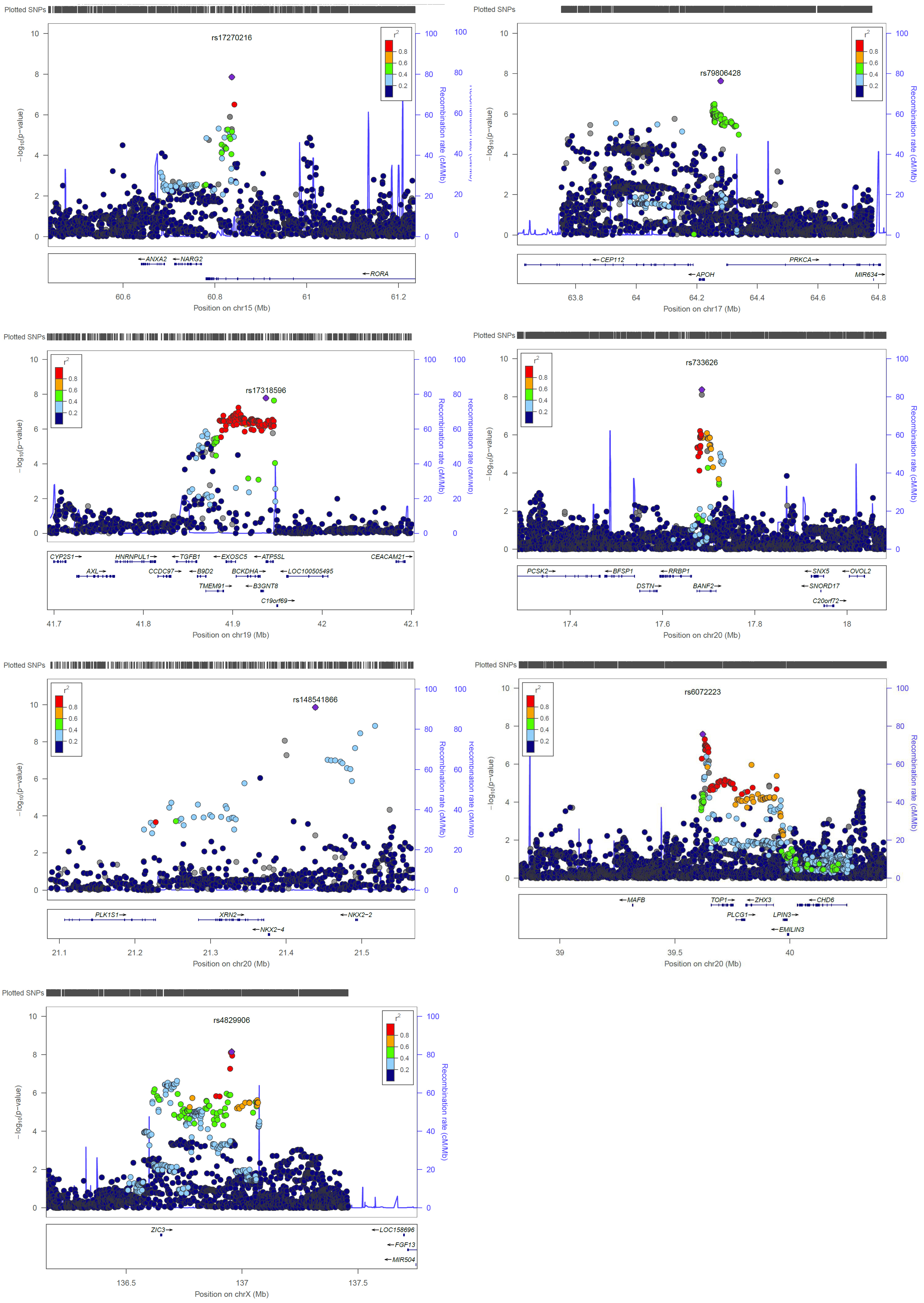
Plotted SNPs
rs17270216
ANXA2
NARG2
RORA
Position on chr15 (Mb)
rs79806428
CEP112
APOH
PRKCA
MIR634
Position on chr17 (Mb)
rs17318596
CYP2S1
AXL
HNRNPUL1
CCDC97
TGFB1
B9D2
TMEM91
EXOSC5
BCKDHA
B3GNT8
ATP5SL
C19orf69
LOC100505495
CEACAM21
Position on chr19 (Mb)
rs733626
PCSK2
BFSP1
DSTN
RRBP1
BANF2
SNX5
SNORD17
C20orf72
OVOL2
Position on chr20 (Mb)
rs148541866
PLK1S1
XRN2
NKX2-4
NKX2-2
Position on chr20 (Mb)
rs6072223
MAFB
TOP1
PLCG1
ZHX3
LPIN3
EMILIN3
CHD6
Position on chr20 (Mb)
rs4829906
ZIC3
LOC158696
FGF13
MIR504
Position on chrX (Mb)
−log10(p−value)
Recombination rate (cM/Mb)
r2
0.8
0.6
0.4
0.2

b

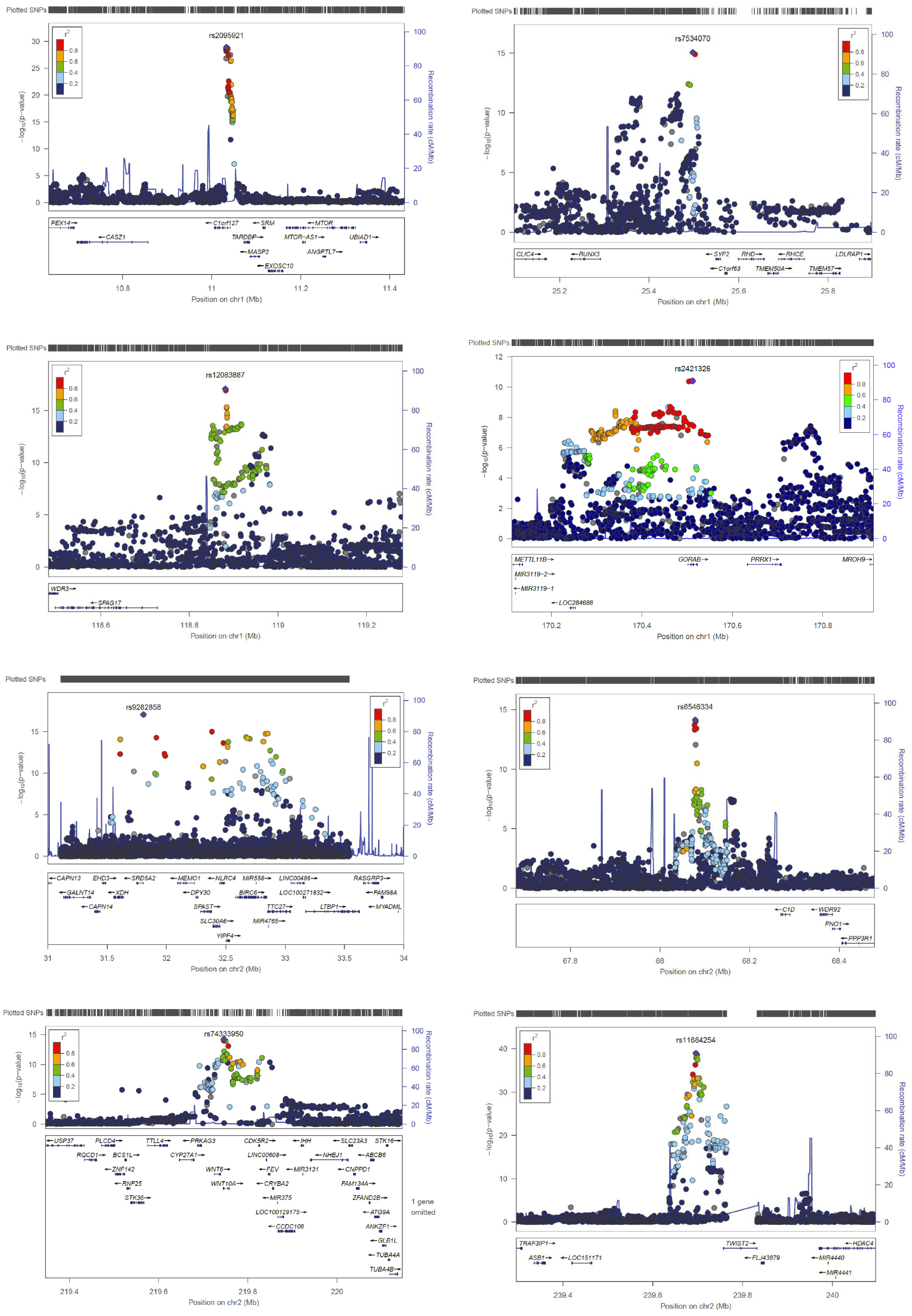

Plotted SNPs
rs2095921
r²
−log₁₀(p−value)
Recombination rate (cM/Mb)
PEX14
CASZ1
C1orf127
SRM
MTOR
TARDBP
MTOR−AS1
UBIAD1
MASP2
ANGPTL7
EXOSC10
Position on chr1 (Mb)
rs7534070
CLIC4
RUNX3
SYF2
RHD
RHCE
LDLRAP1
C1orf63
TMEM50A
TMEM57
Position on chr1 (Mb)
rs12083887
WDR3
SPAG17
Position on chr1 (Mb)
rs2421326
METTL11B
GORAB
PRRX1
MROH9
MIR3119−2
MIR3119−1
LOC284688
Position on chr1 (Mb)
rs9282858
CAPN13
EHD3
SRD5A2
MEMO1
NLRC4
MIR558
LINC00486
RASGRP3
GALNT14
XDH
DPY30
BIRC6
LOC100271832
FAM98A
CAPN14
SPAST
TTC27
LTBP1
MYADML
SLC30A6
MIR4765
YIPF4
Position on chr2 (Mb)
rs6546334
C1D
WDR92
PNO1
PPP3R1
Position on chr2 (Mb)
rs74333950
USP37
PLCD4
TTLL4
PRKAG3
CDK5R2
IHH
SLC23A3
STK16
RQCD1
BCS1L
CYP27A1
LINC00608
NHEJ1
ABCB6
ZNF142
WNT6
FEV
MIR3131
CNPPD1
RNF25
WNT10A
CRYBA2
FAM134A
STK36
MIR375
ZFAND2B
LOC100129175
ATG9A
CCDC108
ANKZF1
GLB1L
TUBA4A
TUBA4B
1 gene omitted
Position on chr2 (Mb)
rs11684254
TRAF3IP1
TWIST2
HDAC4
ASB1
LOC151171
FLJ43879
MIR4440
MIR4441
Position on chr2 (Mb)

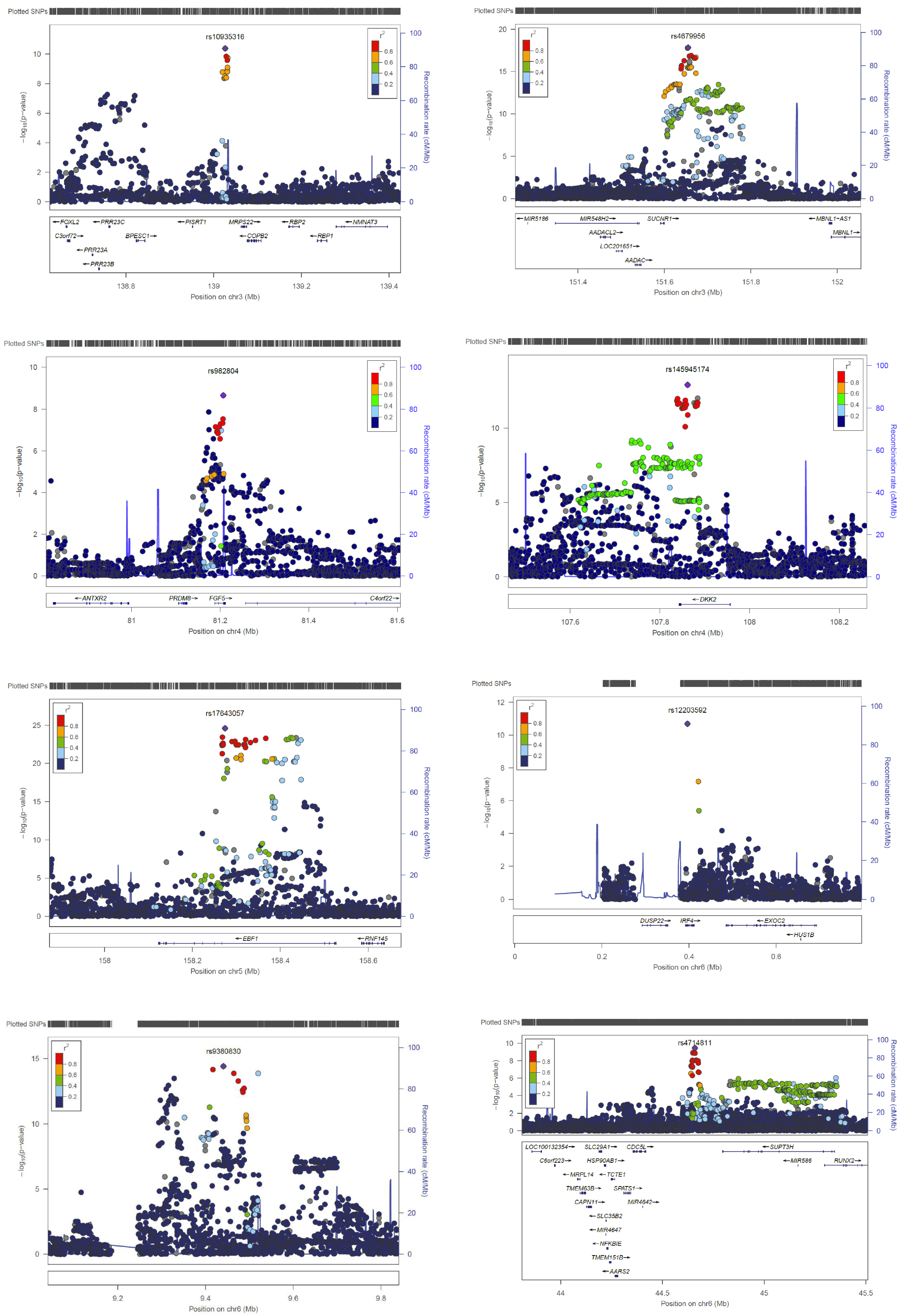

Plotted SNPs
rs10935316
−log10(p−value)
Recombination rate (cM/Mb)
FOXL2
PRR23C
C3orf72
PRR23A
PRR23B
BPESC1
PISRT1
MRPS22
COPB2
RBP2
RBP1
NMNAT3
Position on chr3 (Mb)
rs4679956
MIR5186
MIR548H2
AADACL2
LOC201651
AADAC
SUCNR1
MBNL1-AS1
MBNL1
rs982804
ANTXR2
PRDM8
FGF5
C4orf22
Position on chr4 (Mb)
rs145945174
DKK2
rs17643057
EBF1
RNF145
Position on chr5 (Mb)
rs12203592
DUSP22
IRF4
EXOC2
HUS1B
Position on chr6 (Mb)
rs9380830
rs4714811
LOC100132354
C6orf223
MRPL14
TMEM63B
CAPN11
SLC35B2
MIR4647
NFKBIE
TMEM151B
AARS2
SLC29A1
HSP90AB1
TCTE1
SPATS1
MIR4642
CDC5L
SUPT3H
MIR586
RUNX2

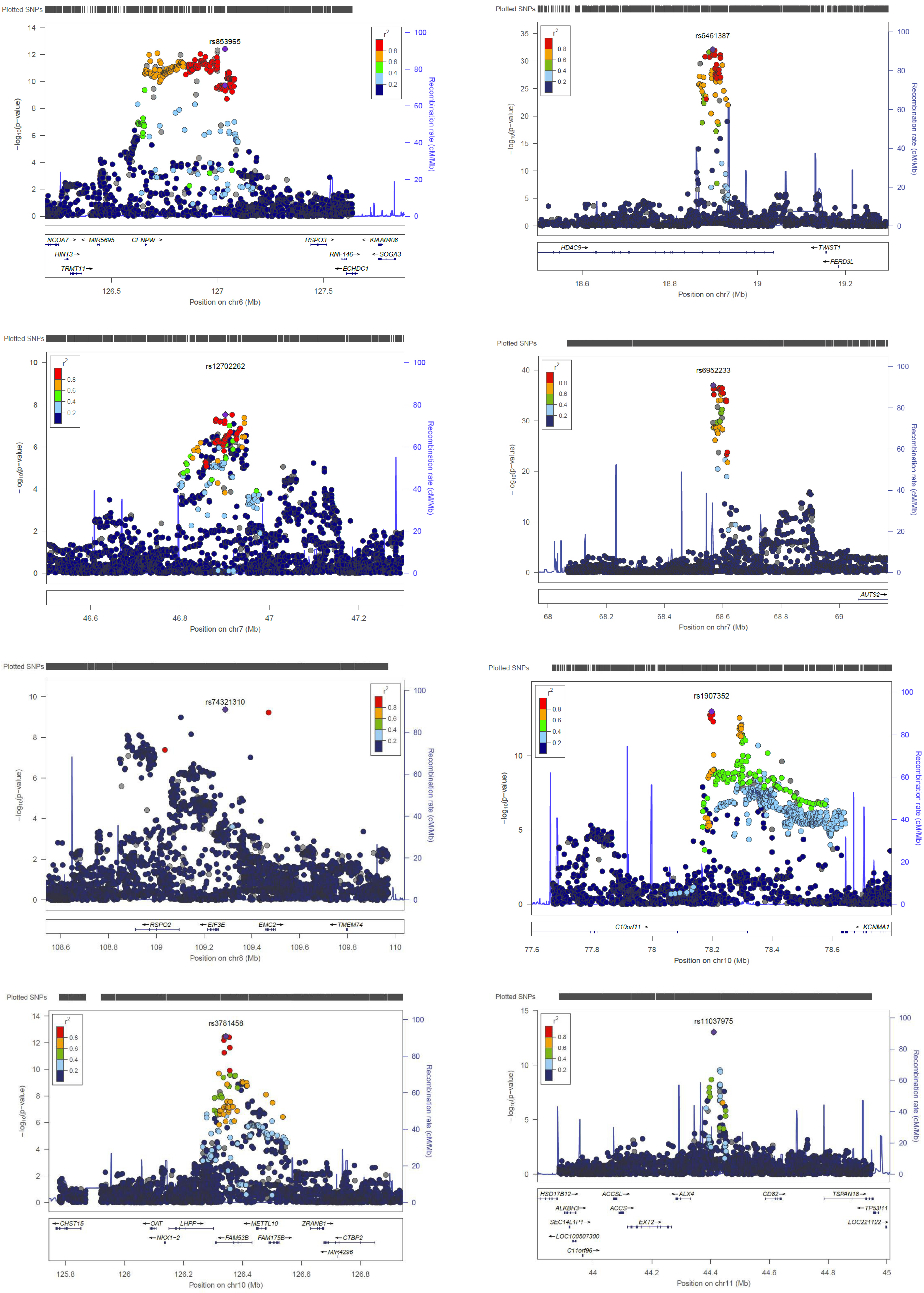
Plotted SNPs
rs853965
Recombination rate (cM/Mb)
NCOA7
MIR5695
CENPW
RSPO3
KIAA0408
HINT3
RNF146
SOGA3
TRMT11
ECHDC1
Position on chr6 (Mb)
rs6461387
HDAC9
TWIST1
FERD3L
Position on chr7 (Mb)
rs12702262
Position on chr7 (Mb)
rs6952233
AUTS2
Position on chr7 (Mb)
rs74321310
RSPO2
EIF3E
EMC2
TMEM74
Position on chr8 (Mb)
rs1907352
C10orf11
KCNMA1
Position on chr10 (Mb)
rs3781458
CHST15
OAT
LHPP
METTL10
ZRANB1
NKX1-2
FAM53B
FAM175B
CTBP2
MIR4296
Position on chr10 (Mb)
rs11037975
HSD17B12
ACCSL
ALX4
CD82
TSPAN18
ALKBH3
ACCS
TP53I11
SEC14L1P1
EXT2
LOC221122
LOC100507300
C11orf96
Position on chr11 (Mb)

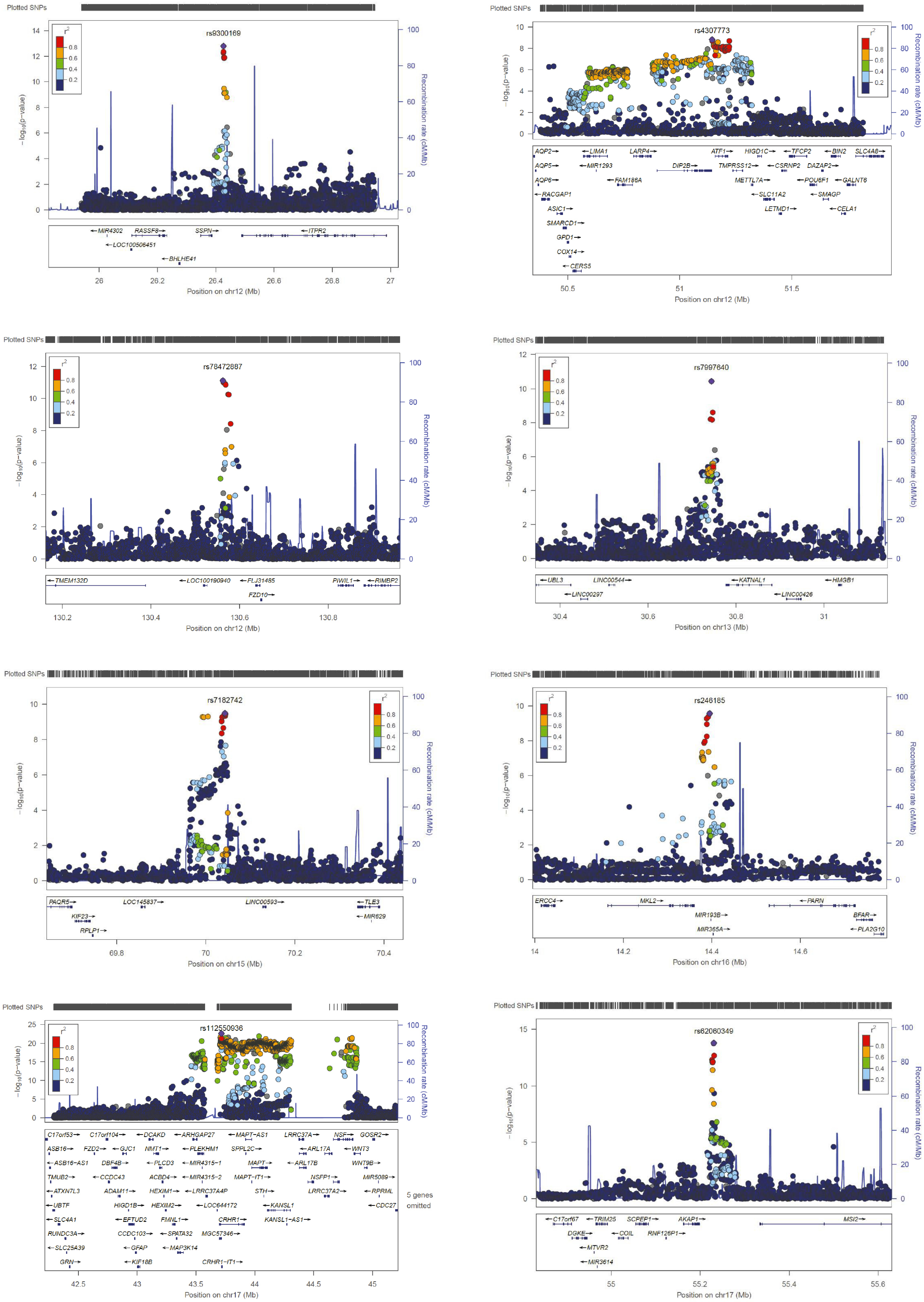

Plotted SNPs
rs9300169
−log10(p−value)
Recombination rate (cM/Mb)
Position on chr12 (Mb)
MIR4302
RASSF8
SSPN
ITPR2
LOC100506451
BHLHE41
rs4307773
AQP2
LIMA1
LARP4
ATF1
HIGD1C
TFCP2
BIN2
SLC4A8
AQP5
MIR1293
DIP2B
TMPRSS12
CSRNP2
DAZAP2
AQP6
FAM186A
METTL7A
POU6F1
GALNT6
RACGAP1
SLC11A2
SMAGP
ASIC1
LETMD1
CELA1
SMARCD1
GPD1
COX14
CERS5
rs78472887
TMEM132D
LOC100190940
FLJ31485
PIWIL1
RIMBP2
FZD10
rs7997640
UBL3
LINC00544
KATNAL1
HMGB1
LINC00297
LINC00426
Position on chr13 (Mb)
rs7182742
PAQR5
LOC145837
LINC00593
TLE3
KIF23
MIR629
RPLP1
Position on chr15 (Mb)
rs246185
ERCC4
MKL2
PARN
MIR193B
BFAR
MIR365A
PLA2G10
Position on chr16 (Mb)
rs112550936
C17orf53
C17orf104
DCAKD
ARHGAP27
MAPT−AS1
LRRC37A
NSF
GOSR2
ASB16
FZD2
GJC1
NMT1
PLEKHM1
SPPL2C
ARL17A
WNT3
ASB16−AS1
DBF4B
PLCD3
MIR4315−1
MAPT
ARL17B
WNT9B
TMUB2
CCDC43
ACBD4
MIR4315−2
MAPT−IT1
NSFP1
MIR5089
ATXN7L3
ADAM11
HEXIM1
LRRC37A4P
STH
LRRC37A2
RPRML
UBTF
HIGD1B
HEXIM2
LOC644172
KANSL1
CDC27
SLC4A1
EFTUD2
FMNL1
CRHR1
KANSL1−AS1
RUNDC3A
CCDC103
SPATA32
MGC57346
SLC25A39
GFAP
MAP3K14
GRN
KIF18B
CRHR1−IT1
5 genes omitted
Position on chr17 (Mb)
rs62060349
C17orf67
TRIM25
SCPEP1
AKAP1
MSI2
DGKE
COIL
RNF126P1
MTVR2
MIR3614

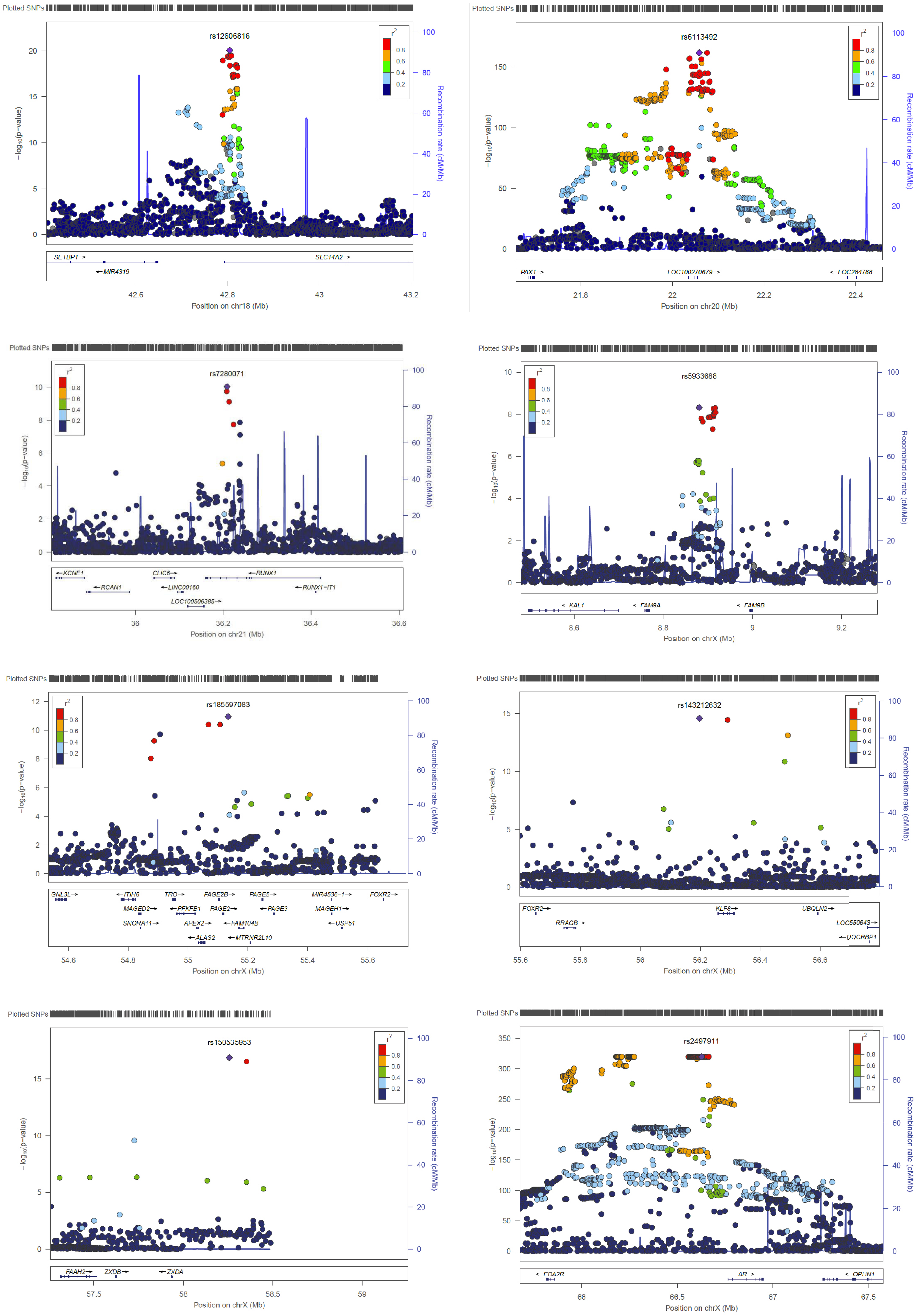
Plotted SNPs
rs12606816
−log10(p−value)
Recombination rate (cM/Mb)
SETBP1→
←MIR4319
SLC14A2→
42.6
42.8
43
43.2
Position on chr18 (Mb)
Plotted SNPs
rs6113492
−log10(p−value)
Recombination rate (cM/Mb)
PAX1→
LOC100270679→
←LOC284788
21.8
22
22.2
22.4
Position on chr20 (Mb)
Plotted SNPs
rs7280071
−log10(p−value)
Recombination rate (cM/Mb)
←KCNE1
←RCAN1
CLIC6→
←LINC00160
LOC100506385→
←RUNX1
←RUNX1−IT1
36
36.2
36.4
36.6
Position on chr21 (Mb)
Plotted SNPs
rs5933688
−log10(p−value)
Recombination rate (cM/Mb)
←KAL1
←FAM9A
←FAM9B
8.6
8.8
9
9.2
Position on chrX (Mb)
Plotted SNPs
rs185597083
−log10(p−value)
Recombination rate (cM/Mb)
GNL3L→
←ITIH6
MAGED2→
SNORA11→
TRO→
←PFKFB1
APEX2→
←ALAS2
PAGE2B→
PAGE2→
←FAM104B
←MTRNR2L10
PAGE5→
←PAGE3
MIR4536−1→
MAGEH1→
←USP51
FOXR2→
54.6
54.8
55
55.2
55.4
55.6
Position on chrX (Mb)
Plotted SNPs
rs143212632
−log10(p−value)
Recombination rate (cM/Mb)
FOXR2→
RRAGB→
KLF8→
UBQLN2→
LOC550643→
←UQCRBP1
55.6
55.8
56
56.2
56.4
56.6
Position on chrX (Mb)
Plotted SNPs
rs150535953
−log10(p−value)
Recombination rate (cM/Mb)
FAAH2→
ZXDB→
←ZXDA
57.5
58
58.5
59
Position on chrX (Mb)
Plotted SNPs
rs2497911
−log10(p−value)
Recombination rate (cM/Mb)
←EDA2R
AR→
←OPHN1
66
66.5
67
67.5
Position on chrX (Mb)

Supplementary Figure 1: Local association plots for the 23 novel (a) and 40 previously known (b) genome-wide significant MPB risk loci. In each panel, the lead SNP is denoted in purple with its rs-ID. The color of the other SNPs indicates the linkage disequilibrium (LD) with lead SNP as red ($0.8<r^2\leq1$), orange ($0.6<r^2\leq0.8$), green ($0.4<r^2\leq10.6$), light blue ($0.2< r^2\leq10.4$), and dark blue ($r^2\leq10.2$).

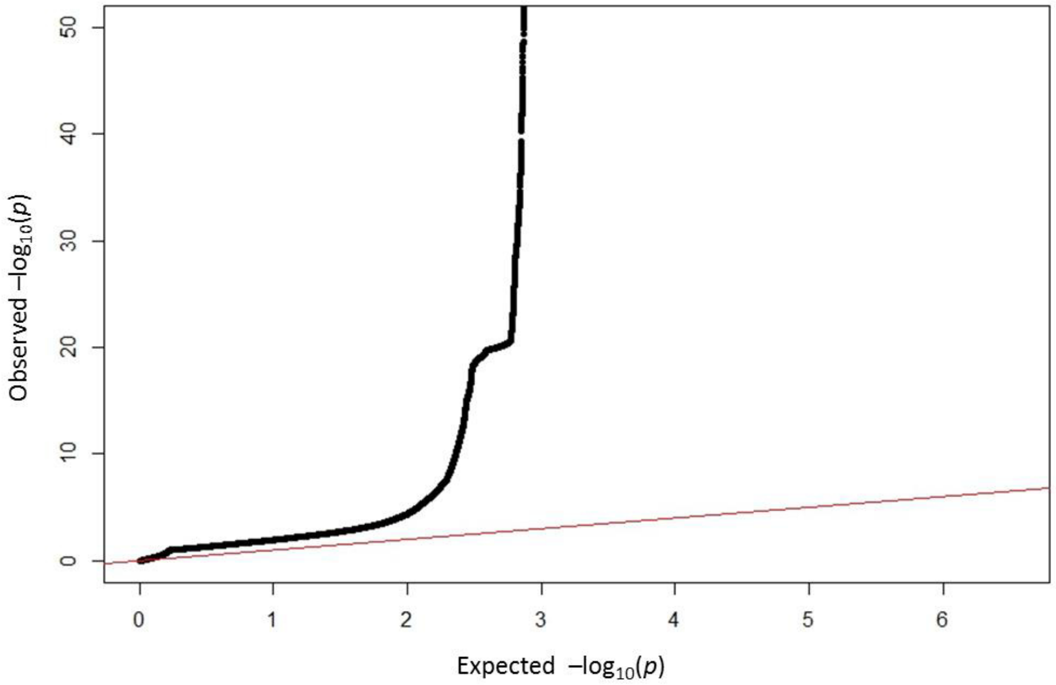

Supplementary Figure 2: Quantile-quantile plot of the meta-analysis. Quantile-quantile plot of the genome-wide meta-analysis of eight case-control samples for male-pattern baldness (MPB) (10,846 cases, 11,672 controls). The x-axis shows the -$\log_{10}$(*p*-values) expected under the null hypothesis. The y-axis shows the observed -$\log_{10}$(*p*-values) derived from the logistic-regression analysis. In line with the observations from meta-analyses of other complex genetic traits that identified high numbers of common risk factors, the test statistic distribution from our meta-analysis deviates from the null hypothesis. This is consistent with previous reports of a polygenetic contribution to MPB[13,14]. Data points with $P<1.0x10^{-50}$ were truncated.

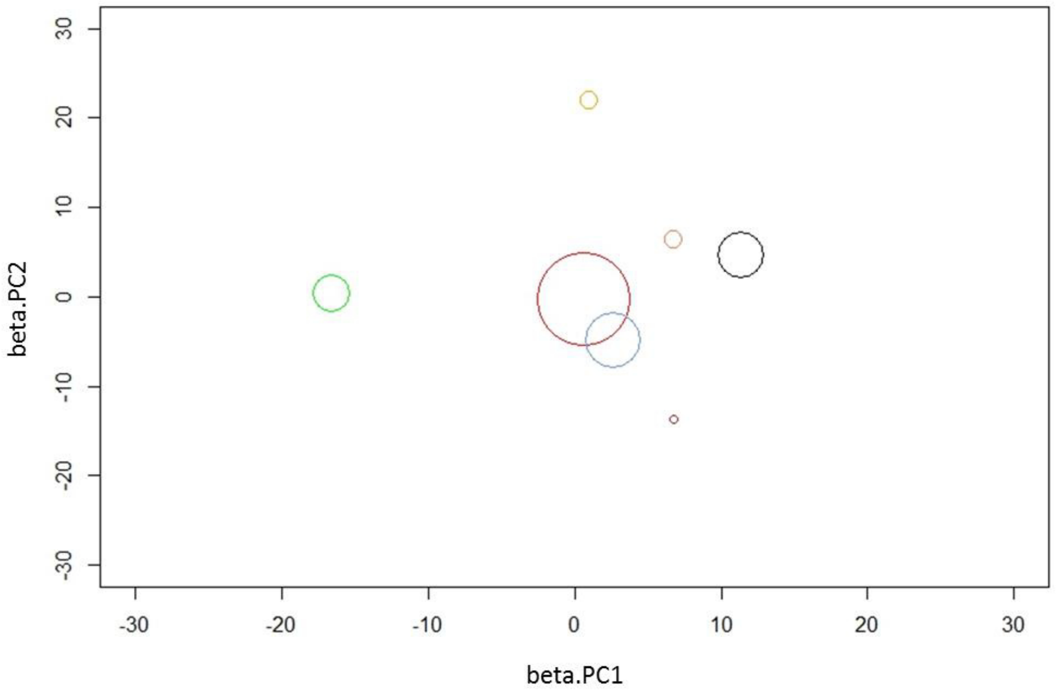

Supplementary Figure 3: Homogeneity of effects across the eight studies. Across-study homogeneity plot of the first two principal components (PCs) of the logistic regression β coefficients for 52 genome-wide significant associations from 7 of the eight studies (23andMe, red; Bonn, green; Nijmegen, orange; QIMRB_1, blue; QIMRB_2, coral; THISEAS, yellow; and TwinUK, black).The CoLaus Cohort has been omitted due to missing values for 26 SNPs). PCs are weighted by the number of cases. Each circle shows the location of a study on PC1 and PC2.Circle sizes are proportional to the number of cases in each sample. Samples cluster around the 23andMe cohort. Effect sizes in the Bonn samples were generally larger (average absolute β value increase of 75%). This can be attributed to the extreme sampling approach and the exact phenotyping by dermatology specialists (severely affected early-onset cases, elderly unaffected controls) used in the Bonn study, as compared to the self-reporting approach applied in the 23andMe cohort.

Supplementary Tables

Supplementary Table 1: Summary of the demographic properties, sample sizes and study specific genotyping platforms, imputation methods and genome wide association methods in the eight participant studies.

Study	sample description				Genotyping & Imputation				Meta analysis			
	country of origin	descent	study type	sample size (cases/controls)	Platform	Genotype calling algorithm	Imputation software	Reference Panel	Inclusion criteria: MAF	Inclusion criteria: Imputation quality	SNPs in meta-analysis	λ_{gc}
23andMe	USA	European	Cohort (population based)	17,500 (9,009/ 8,491)	Illumina 550+	Illumina GenomeStudio	BEAGLE /minimac	1K	0.001	0.5	13,426,705	1.095
Bonn	Germany	Germany	Case-control (population based)	997 (581/ 416)	Illumina 317K, 317Kduo, 550K, 610K	Illumina BeadStudio/ Genome Studio	IMPUTE	1K	0.01	0.4	8,629,219	1.038
CoLaus	Switzerland	European	Cohort (community sample)	1,277 (622/ 655)	Affymetrix GeneChip Human Mapping 500K	BRLMM algorithm	IMPUTE	1K	0.01	0.4	12,502,202	1.019
Nijmegen	Netherlands	European	Cohort (population based)	392 (145/ 247)	Illumina CNV370k	Illumina BeadStudio	IMPUTE	1K	0.01	0.4	9,185,630	1.014
QIMRB1	Australia	European	Cohort (population based)	1,378 (216/ 1,162)	Illumina CNV370K, 610K	Illumina BeadStudio/ Genome Studio	MACH	1K	0.01	0.3	8,854,429	1.020
QIMRB2	Australia	European	Cohort (population based)	400 (59/ 341)	Illumina HumanCoreExome	Illumina BeadStudio/ Genome Studio	MACH	1K	0.01	0.3	8,671,470	0.999
THISEAS	Greece	European	Case-control (population based)	202 (52/ 150)	MetaboChip	GenoSNP	IMPUTE	1K	0.01	0.4	11,755,623	1.030
TwinsUK	UK	UK	Cohort (population based)	372 (162/ 210)	Illumina 610K	Illuminus	IMPUTE	1K	0.01	0.4	8,593,674	1.005
				**22,518 (10,846/11,672)**								

1KG -1000Genomes

Supplementary Table 2: Overlap between MPB risk loci and eQTL findings from blood[15], skin[16] (dbGaP Accession phs000424.v6.p1) and hair follicle.

eSNP	r^2	eSNP Chr	eSNP Position	eGenes	Distance between MPB SNP and eSNP	Tissue
rs9430158	0.7	1	11043861	*SRM*	10539	Blood
rs6678044	0.5	1	25487889	*RHD,TMEM50A*	10286	Blood
**rs17371253**	**1.0**	**1**	**47955427**	***CMPK1***	**0**	**Blood**
rs12735613	0.5	1	118883973	*ROBO1*	2284	Blood
**rs17185996**	**1.0**	**1**	**119608732**	***WARS2***	**0**	**Blood**
rs1333141	0.5	1	170407926	*GORAB*	104443	Blood
rs4669067	0.7	2	6541524	-	12176	Blood
rs4574113	0.5	2	219762662	*CYP27A1*	16370	Blood
rs7349332	0.9	2	219756383	-	10091	Blood
rs9846116	0.5	3	126083378	*CHST13*	14599	Blood
rs10935880	0.6	3	151655279	*SUCNR1*	417	Blood
**rs4679956**	**1.0**	**3**	**151654862**	***SUCNR1***	**0**	**Whole_Blood**
rs3733336	0.7	4	81207963	*PRDM8*	924	Blood
rs10447201	0.5	5	158418735	*RNF145*	144244	Blood
**rs12203592**	**1.0**	**6**	**396321**	***EXOC2 (cis), AC133555.3-2,SULT1A4,GIYD1 (trans)***	**0**	**Blood**
**rs12203592**	**1.0**	**6**	**396321**	***IRF4***	**0**	**Whole_Blood**
rs12529522	0.5	6	44865520	*SUPT3H*	205934	Blood
rs12702255	0.9	7	46878747	*AC011294.3*	22547	Skin_Not_Sun_Exposed_Suprapubic
rs3781476	0.5	10	126314475	*FAM53B,LHPP*	29456	Blood
rs2362515	0.7	10	126336158	*FAM53B,LHPP,OAT*	7773	Blood

rs3781470	0.8	10	126319992	*FAM53B,OAT*	23939	Blood
**rs3781458**	**1.0**	**10**	**126343931**	***FAM53B***	**0**	**Blood**
rs7306330	0.5	12	51320290	*DIP2B,TFCP2,METTL7A,ATF1*	175858	Blood
rs7136702	0.5	12	50880216	*ATF1*	264216	Skin_Sun_Exposed_Lower_leg
rs12422417	0.6	12	50764711	*LASS5,LIMA1*	379721	Blood
rs4348979	0.6	12	50763415	*LASS5,LIMA1,C12orf62*	381017	Blood
rs2280503	0.7	12	51138687	*DIP2B*	5745	Hair follicle
rs7489214	0.7	12	50723993	*LASS5,LIMA1,C12orf62,SMARCD1*	420439	Blood
rs11169449	0.7	12	50882355	*CERS5*	262077	Skin_Not_Sun_Exposed_Suprapubic
rs11169477	0.7	12	50919297	*CERS5*	225135	Skin_Sun_Exposed_Lower_leg
rs10747583	0.7	12	50946955	*DIP2B*	197477	Blood
rs12427378	0.7	12	51074199	*DIP2B,ATF1*	70233	Blood
rs11503907	0.9	12	51153577	*DIP2B*	9145	Whole_Blood
**rs4307773**	**1.0**	**12**	**51144432**	***DIP2B,ATF1,METTL7A***	**0**	**Blood**
**rs4307773**	**1.0**	**12**	**51144432**	***ATF1,CERS5***	**0**	**Skin_Sun_Exposed_Lower_leg**
rs56393023	0.5	15	57528046	*LINC00926*	22918	Whole_Blood
rs8042606	0.6	15	57100213	*ZNF280D*	450751	Blood
**rs7173477**	**1.0**	**15**	**57550964**	***LINC00926***	**0**	**Skin_Sun_Exposed_Lower_leg**
rs17191463	0.5	15	60824601	*NARG2,-*	12193	Blood
**rs17270216**	**1.0**	**15**	**60836794**	***ANXA2***	**0**	**Blood**
rs7176508	0.6	15	70018990	*IGLL1,GPR144*	23898	Blood
rs1659127	0.9	16	14388305	*PAX8*	7127	Blood
**rs112550936**	**1.0**	**17**	**43716155**	***DND1P1,CRHR1,RP11-259G18.3,LRRC37A4P,RP11-707O23.5,CRHR1-IT1,RPS26P8,KANSL1-AS1,LRRC37A,RP1***	**0**	**Skin_Sun_Exposed_Lower_leg**

				1-259G18.1,RP11-259G18.2,LRRC37A2,ARHGAP27		
**rs112550936**	**1.0**	**17**	**43716155**	***RPS26P8,DND1P1,KANSL1-AS1,LRRC37A,CRHR1,RP11-259G18.1,RP11-259G18.3,LRRC37A4P,RP11-259G18.2,LRRC37A2,ARHGAP27,RP11-707O23.5,CRHR1-IT1***	**0**	**Skin_Not_Sun_Exposed_Suprapubic**
**rs112550936**	**1.0**	**17**	**43716155**	***DND1P1,CRHR1,RP11-798G7.5,RP11-259G18.3,LRRC37A4P,RP11-707O23.5,CRHR1-IT1,KANSL1-AS1,LRRC37A,RP11-259G18.2,LRRC37A2***	**0**	**Whole_Blood**
rs11879090	0.5	19	41917260	*BCKDHA,B3GNT8,B9D2,EXOSC5*	19835	Blood
rs284653	0.5	19	41928701	*B3GNT8,TMEM91*	8394	Skin_Sun_Exposed_Lower_leg
rs284653	0.5	19	41928701	*TMEM91*	8394	Whole_Blood
rs4674	0.9	19	41930396	*CTC-435M10.6*	6699	Whole_Blood
rs2569400	0.9	19	41936608	*ATP5SL,CTC-435M10.6*	487	Whole_Blood
rs1043413	0.9	19	41939297	*ATP5SL*	2202	Whole_Blood
rs2231940	0.9	19	41944237	*B3GNT8*	7142	Skin_Sun_Exposed_Lower_leg
**rs17318596**	**1.0**	**19**	**41937095**	***BCKDHA,B3GNT8,B9D2***	**0**	**Blood**
rs6075239	0.5	20	17689080	*RRBP1*	3731	Blood
rs6080782	0.5	20	17674599	*DSTN,RRBP1*	10750	Blood
rs3194551	0.5	20	17680672	*RRBP1,PCSK2*	4677	Blood

rs6080791	1.0	20	17683799	*RRBP1,DSTN*	1550	Blood
rs6124298	0.5	20	39617398	*ZHX3,TOP1*	3449	Blood
rs151147906	0.8	20	39835512	*LPIN3*	214665	Skin_Sun_Exposed_Lower_leg
rs1006325	1.0	20	39633603	*TOP1*	12756	Blood

MPB lead SNPs that have been associated with the expression of one or more genes are in bold

.

Supplementary Table 3: List of miRNA genes and miRNA target genes at MPB risk loci.

Chromosomal Region	lead SNP	Region Chr:Start-Stop	miRNA Gene	strand	miRNA Chr:Start-End	Expressed in human hair follicle	Target Genes at MPB loci (+- 500kb)
1p36.11	rs7534070	1:25,318,225-25,511,358	*MIR6731*	-	*chr1:25245835-25245907*	no	
1p36.11	rs7534070	1:25,318,225-25,511,358	*MIR4425*	+	*chr1:25349993-25350077*	no	
1q24.2	rs2421326	1:170,229,218-170,546,697	*MIR3119-2*	+	*chr1:170120518-170120603*	no	
1q24.2	rs2206310	1:170,712,490-170,791,811	*MIR1295A*	-	*chr1:171070869-171070947*	no	
1q24.2	rs2206310	1:170,712,490-170,791,811	*MIR1295B*	+	*chr1:171070879-171070939*	no	
2p25.2	rs12997617	2:6,458,933-6,573,741	*MIR7158*	+	*chr2:6114793-6114864*	no	
2p25.2	rs12997617	2:6,458,933-6,573,741	*MIR7515*	+	*chr2:6790504-6790571*	**yes**	
2q31.1	rs1819008	2:177,588,381-177,796,823	*MIR6512*	-	*chr2:178178533-178178610*	no	
2q31.1	rs1819008	2:177,588,381-177,796,824	*MIR1246*	-	*chr2:177465707-177465780*	**yes**	*ATF1, RORA*
2q31.1	rs1819008	2:177,588,381-177,796,825	*MIR4444-1*	+	*chr2:178077453-178077526*	**yes**	
2q31.1	rs1819008	2:177,588,381-177,796,825	*MIR4444-2*	+	*chr2:178077453-178077526*	**yes**	
2q31.1	rs1819008	2:177,588,381-177,796,826	*MIR3128*	-	*chr2:178120672-178120738*	**yes**	
2q35	rs74333950	2:219,691,566-219,854,317	*MIR9500*	+	*chr2:219687812-219687877*	no	
2q35	rs74333950	2:219,691,566-219,854,317	*MIR3131*	-	*chr2:219923409-219923472*	no	
2q35	rs74333950	2:219,691,566-219,854,317	*MIR26B*	+	*chr2:219267368-219267445*	**yes**	*BFAR, CEACAM4, CMPK1, CSRP2BP, CYP2F1, DDX60, DUSP22, EXT2, FMO3, HSD3B1, NFKBIE, NKX2-2, PET117, RCAN1, RGS3, RPLP1, RSAD2, SLC41A3, SUPT3H, TCF12, TOP1, WDR92, XDH CHST15, STIL*
2q35	rs74333950	2:219,691,566-219,854,317	*MIR375*	-	*chr2:219866366-219866430*	**yes**	*AXL, RRAGB, TNS3, UBE3A*
2q35	rs74333950	2:219,691,566-219,854,317	*MIR153-1*	-	*chr2:220158832-220158922*	**yes**	*NFE2L2, POLE3*
2q37.3	rs11684254	2:239,638,300-239,764,032	*MIR4440*	-	*chr2:239990512-239990610*	**yes**	
2q37.3	rs11684254	2:239,638,300-239,764,032	*MIR4441*	-	*chr2:240007522-240007622*	**yes**	*PCSK2, SLC29A1*
3q25.1	rs4679956	3:151,599,393-151,781,175	*MIR548H2*	+	*chr3:151347319-151542394*	**yes**	

6p21.1	rs4714811	6:44,639,723-44,676,420	*MIR4647*	-	*chr6:44221942-44222022*	no	
6p21.1	rs4714811	6:44,639,723-44,676,420	*MIR4642*	+	*chr6:44403377-44403459*	no	
6q22.32	rs144578168	6:126,609,124-127,137,371	*MIR588*	+	*chr6:126805776-126805859*	no	
9q32	rs201655553	9:116,511,364-116,691,072	*MIR455*	+	*chr9:116971713-116971809*	**yes**	*CDC5L, CLIC4, CYP2B6, DAZAP2, DGKE, GNL3L, HSP90AB1, KATNAL1, RHD, ERCC4*
10q26.13	rs3781458	10:126,278,648-126,537,000	*MIR4296*	-	*chr10:126721351-126721439*	**yes**	*PRL, TMEM50A*
12p12.1	rs9300169	12:26,438,616-26,446,289	*MIR4302*	-	*chr12:26026952-26027012*	no	
15q12	rs4906800	15:26,120,550-26,121,173	*MIR4715*	-	*chr15:26093893-26093972*	**yes**	*C10orf11, LIMA1*
15q23	rs7182742	15:69,992,647-70,048,157	*MIR629*	-	*chr15:70371710-70371807*	**yes**	*ANTXR2, FAM175B, HNRNPUL1*
16p13.12	rs246185	16:14,377,400-14,406,119	*MIR193B*	+	*chr16:14397823-14397906*	**yes**	*BCKDHA, HMGB1, KLHL42, PPA2, PRKCA, RPS19, UBQLN2, ZNF618*
16p13.12	rs246185	16:14,377,400-14,406,119	*MIR365A*	+	*chr16:14403141-14403228*	**yes**	*ALDH1L1, CEACAM5, UBL3, ZXDA*
17q21.31	rs112550936	17:43,292,035-44,877,887	*MIR4315-2*	-	*chr17:43552728-43552801*	no	
17q21.31	rs112550936	17:43,292,035-44,877,887	*MIR4315-1*	-	*chr17:43552728-43552801*	no	
17q22	rs62060349	17:55,226,801-55,237,542	*MIR3614*	-	*chr17:54968630-54968716*	no	
18q12.3	rs12606816	18:42,669,587-42,829,042	*MIR4319*	-	*chr18:42550046-42550131*	**yes**	*ACCSL, C1D, IRF4, NOTCH2*

Supplementary Table 4: Results of the DEPICT candidate gene priorization at the 63 MPB risk loci (P<0.05)

Region (Genome build GRCh37)	Cytogenetic band	# Genes in locus	Gene ID	Gene symbol	*P* value	FDR
chr2:31557187-33624576	2p23.1	11	ENSG00000049319	*SRD5A2*	2.26E-04	≤ 0.20
chr6:391739-411447	6p25.3	1	ENSG00000137265	*IRF4*	2.86E-04	≤ 0.20
chr1:25226002-25291612	1p36.11	1	ENSG00000020633	*RUNX3*	1.41E-03	≤ 0.20
chr6:9596343-10211841	6p24.3	1	ENSG00000181355	*OFCC1*	1.77E-03	≤ 0.20
chr15:60771377-61521518	15q22.2	2	ENSG00000069667	*RORA*	6.01E-03	> 0.20
chr1:170904612-171033906	1q24.2	1	ENSG00000117501	*C1orf129*	6.18E-03	> 0.20
chr15:70127573-70135459	15q23	1	ENSG00000259703	*C15orf50*	6.27E-03	> 0.20
chr19:41856816-42006550	19q13.2	4	ENSG00000177191	*B3GNT8*	6.64E-03	> 0.20
chr12:50569571-51214905	12q13.12	5	ENSG00000123268	*ATF1*	7.23E-03	> 0.20
chr21:36160098-37357047	21q22.12	1	ENSG00000159216	*RUNX1*	7.64E-03	> 0.20
chr13:30855902-30856006	13q12.3	1	ENSG00000252928	*RNU6-64*	7.88E-03	> 0.20
chr17:43471275-44896082	17q21.31	12	ENSG00000204650	*CRHR1-IT1*	9.58E-03	> 0.20
chr4:169013666-169108893	4q32.3	1	ENSG00000109511	*ANXA10*	0.01	> 0.20
chr5:158122924-158526788	5q33.3	1	ENSG00000164330	*EBF1*	0.01	> 0.20
chr7:46820971-46823041	7p12.3	1	ENSG00000242948	*EPS15P1*	0.01	> 0.20
chr20:39652845-39995467	20q12	7	ENSG00000198900	*TOP1*	0.01	> 0.20
chr1:119425666-119818596	1p12	3	ENSG00000092607	*TBX15*	0.02	> 0.20
chr6:128029217-128239776	6q22.33	1	ENSG00000172673	*THEMIS*	0.02	> 0.20
chr15:56922379-57599965	15q21.3	3	ENSG00000137871	*ZNF280D*	0.02	> 0.20
chr2:6779255-6790651	2p25.2	1	ENSG00000236172	*MIR7515HG*	0.03	> 0.20
chr2:176986339-177001826	2q31.1	1	ENSG00000237380	*HOXD-AS2*	0.03	> 0.20
chr2:31557187-33624576	2p23.1	11	ENSG00000230876	*LINC00486*	0.04	> 0.20

chr2:219745085-219826876	2q35	3	ENSG00000228135	*LINC01494*	0.04	> 0.20
chr4:106067032-106200973	4q24	1	ENSG00000168769	*TET2*	0.04	> 0.20

FDR – false discovery rate

Supplementary Table 5: Top 20 nominally significant results from the DEPICT analysis of gene enrichment at MPB associated loci in specific gene-sets

Gene set ID	Gene set description	*P* value	FDR
MP:0001759	increased urine glucose level	1.16E-05	> 0.20
GO:0031056	regulation of histone modification	4.35E-05	> 0.20
GO:0006342	chromatin silencing	7.85E-05	> 0.20
MP:0001859	kidney inflammation	1.17E-04	> 0.20
MP:0002357	abnormal spleen white pulp morphology	1.35E-04	> 0.20
MP:0000492	abnormal rectum morphology	1.56E-04	> 0.20
ENSG00000071655	MBD3 PPI subnetwork	1.66E-04	> 0.20
GO:0045814	negative regulation of gene expression, epigenetic	1.76E-04	> 0.20
GO:0051851	modification by host of symbiont morphology or physiology	2.17E-04	> 0.20
GO:0051054	positive regulation of DNA metabolic process	2.25E-04	> 0.20
GO:0042612	MHC class I protein complex	2.36E-04	> 0.20
MP:0008478	increased spleen white pulp amount	2.78E-04	> 0.20
GO:0042035	regulation of cytokine biosynthetic process	2.79E-04	> 0.20
GO:0051702	interaction with symbiont	2.88E-04	> 0.20
MP:0001807	decreased IgA level	4.13E-04	> 0.20
MP:0001852	conjunctivitis	4.84E-04	> 0.20
GO:0051052	regulation of DNA metabolic process	4.96E-04	> 0.20
GO:0033044	regulation of chromosome organization	4.96E-04	> 0.20
GO:0004175	endopeptidase activity	6.62E-04	> 0.20
GO:0001817	regulation of cytokine production	7.38E-04	> 0.20

FDR – false discovery rate

Supplementary Table 6: Results of the DEPICT analysis for an enrichment of genes at MPB associated loci in specific tissue types. The table lists all gene sets that showed a P-value <$5x10^{-3}$.

Tissue type	MeSH Term	MeSH first level term	MeSH second level term	*P* value	FDR
Skeleton	A02.835	Musculoskeletal System	Skeleton	3.98E-05	≤ 0.20
Connective Tissue	A10.165	Tissues	Connective Tissue	4.02E-05	≤ 0.20
Bone and Bones	A02.835.232	Musculoskeletal System	Skeleton	4.83E-05	≤ 0.20
Immune System	A15.382	Hemic and Immune Systems	Immune System	5.68E-05	≤ 0.20
Bone Marrow	A15.382.216	Hemic and Immune Systems	Immune System	6.79E-05	≤ 0.20
Pharynx	A14.724	Stomatognathic System	Pharynx	6.85E-04	≤ 0.20
Blood	A15.145	Hemic and Immune Systems	Blood	8.60E-04	≤ 0.20
Leukocytes Mononuclear	A15.145.229.637.555	Hemic and Immune Systems	Blood	1.02E-03	≤ 0.20
Blood Cells	A15.145.229	Hemic and Immune Systems	Blood	1.06E-03	≤ 0.20
Fetal Blood	A15.145.300	Hemic and Immune Systems	Blood	1.08E-03	≤ 0.20
Leukocytes	A11.118.637	Cells	Blood Cells	1.11E-03	≤ 0.20
Monocytes	A15.378.316.580	Hemic and Immune Systems	Hematopoietic System	2.04E-03	≤ 0.20
Antigen Presenting Cells	A11.066	Cells	Antigen-Presenting Cells	2.77E-03	≤ 0.20
Dendritic Cells	A15.382.812.260	Hemic and Immune Systems	Immune System	2.77E-03	≤ 0.20
Mononuclear Phagocyte System	A15.382.812	Hemic and Immune Systems	Immune System	2.81E-03	≤ 0.20
Lymphocytes	A15.382.490.555.567	Hemic and Immune Systems	Immune System	4.08E-03	≤ 0.20
Oropharynx	A04.623.603	Respiratory System	Pharynx	4.16E-03	≤ 0.20
Palatine Tonsil	A15.382.520.604.800	Hemic and Immune Systems	Immune System	4.16E-03	≤ 0.20

Supplementary Table 7: Results of the Ingenuity Pathway-bases Analysis (IPA).

Ingenuity Canonical Pathways	Ratio	*P (*FE)	P-value(BH)	Molecules
Nicotine Degradation II	0.17	$7.08x10^{-7}$	$4.47x10^{-4}$	*FMO3,CYP2F1,EXT2,FMO2,CYP4X1,CYP2A6 (includes others),FMO1,CYP2B6,CYP2S1*
Bupropion Degradation	0.21	$8.13x10^{-5}$	0.01	*CYP2F1,CYP4X1,CYP2A6 (includes others),CYP2B6,CYP2S1*
Acetone Degradation I (to Methylglyoxal)	0.19	$1.23x10^{-4}$	0.01	*CYP2F1,CYP4X1,CYP2A6 (includes others),CYP2B6,CYP2S1*
Nicotine Degradation III	0.13	$2.75x10^{-4}$	0.03	*CYP2F1,EXT2,CYP4X1,CYP2A6 (includes others),CYP2B6,CYP2S1*
Melatonin Degradation I	0.12	$3.80x10^{-4}$	0.03	*CYP2F1,EXT2,CYP4X1,CYP2A6 (includes others),CYP2B6,CYP2S1*
Superpathway of Melatonin Degradation	0.11	$6.46x10^{-4}$	0.03	*CYP2F1,EXT2,CYP4X1,CYP2A6 (includes others),CYP2B6,CYP2S1*
**Estrogen Biosynthesis**	**0.14**	**$6.76x10^{-4}$**	0.03	***CYP2F1,CYP4X1,CYP2A6 (includes others),CYP2B6,CYP2S1***
**Androgen Biosynthesis**	**0.25**	**0.001**	0.06	***SRD5A2,HSD3B1,HSD3B2***
Retinoate Biosynthesis II	0.50	0.002	0.06	*RBP2,RBP1*
Heparan Sulfate Biosynthesis (Late Stages)	0.10	0.002	0.06	*AADAC,EXT2,GLCE,CHST13,CHST15*
**Adipogenesis pathway**	0.06	0.003	0.07	*FZD10,HDAC9,EBF1,HDAC4,TGFB1,CTBP2,RBP1,PER2*
Tetrapyrrole Biosynthesis II	0.40	0.004	0.08	*ALAS2,ALAD*
Heparan Sulfate Biosynthesis	0.09	0.004	0.08	*AADAC,EXT2,GLCE,CHST13,CHST15*
Xenobiotic Metabolism Signaling	0.05	0.004	0.08	*MAP3K14,ALDH1L1,FMO3,HDAC4,HSP90AB1,FMO2,FMO1,CYP2B6,CHST13,NFE2L2,CHST15,PRKCA*
Mineralocorticoid Biosynthesis	0.29	0.007	0.22	*HSD3B1,HSD3B2*
Role of NFAT in Cardiac Hypertrophy	0.05	0.007	0.09	*HDAC9,RCAN1,HDAC4,TGFB1,ITPR2,PPP3R1,PLCG1,PLCD4,PRKCA*
Glucocorticoid Biosynthesis	0.25	0.010	0.22	*HSD3B1,HSD3B2*
PI3K Signaling in B Lymphocytes	0.06	0.01	0.14	*ATF1,ITPR2,PPP3R1,NFKBIE,PLCG1,CD79A,PLCD4*
Antioxidant Action of Vitamin C	0.06	0.01	0.15	*NFKBIE,PLA2G10,PLCG1,PLCD4,SLC23A3,TXNRD3*
Heme Biosynthesis II	0.22	0.01	0.18	*ALAS2,ALAD*
Retinol Biosynthesis	0.10	0.02	0.23	*AADAC,RBP2,RBP1*
**Wnt/Ca+ pathway**	**0.07**	**0.02**	0.22	***FZD10,PLCG1,PLCD4,PRKCA***

Glioblastoma Multiforme Signaling	0.05	0.02	0.22	*FZD10,MTOR,WNT10A,ITPR2,PLCG1,WNT6,PLCD4*
**EGF Signaling**	**0.07**	**0.02**	0.22	***MTOR,ITPR2,PLCG1,PRKCA***
Role of Macrophages, Fibroblasts and Endothelial Cells in Rheumatoid Arthritis	0.04	0.02	0.22	*MAP3K14,FZD10,WNT10A,TGFB1,PPP3R1,NFKBIE,DKK2,PLCG1,WNT6,PLCD4,PRKCA*
Regulation of the Epithelial-Mesenchymal Transition Pathway	0.04	0.03	0.22	*FZD10,NOTCH2,TWIST2,WNT10A,TGFB1,TWIST1,WNT6,FGF5*
Calcium-induced T Lymphocyte Apoptosis	0.07	0.03	0.27	*ITPR2,PPP3R1,PLCG1,PRKCA*
p70S6K Signaling	0.05	0.03	0.22	*MTOR,MAPT,PLCG1,CD79A,PLCD4,PRKCA*
PCP pathway	0.06	0.03	0.27	*FZD10,RSPO3,WNT10A,WNT6*
The Visual Cycle	0.13	0.03	0.28	*RBP2,RBP1*
Phospholipase C Signaling	0.04	0.04	0.27	*HDAC9,HDAC4,ITPR2,PPP3R1,PLA2G10,PLCG1,CD79A,ARHGEF1,PRKCA*
Spermidine Biosynthesis I	0.50	0.04	0.30	*SRM,*
PPARα/RXRα Activation	0.04	0.04	#NV	*MAP3K14,HSP90AB1,TGFB1,NFKBIE,PLCG1,PLCD4,PRKCA*
**Wnt/beta-catenin Signaling**	**0.04**	**0.04**	#NV	***SOX4,FZD10,WNT10A,TGFB1,TLE3,DKK2,WNT6***
Melatonin Signaling	0.06	0.04	0.30	*RORA,PLCG1,PLCD4,PRKCA*
Acute Phase Response Signaling	0.04	0.04	0.28	*MAP3K14,MTOR,APOH,NFKBIE,AMBP,RBP2,RBP1*
Basal Cell Carcinoma Signaling	0.06	0.04	0.30	*FZD10,STK36,WNT10A,WNT6*

ratio - number of genes in the pathway that map to MPB risk loci/ total number of genes assigned to pathway; P-value (FE) - P-value of right-tailed Fisher's Exact test; P-value (BH) - Benjamini Hochberg corrected P-value

Supplementary Table 8: Comparison of the association signals for the 63 lead SNPs between the complete meta-analysis (MA), the 23andMe cohort (23andMe), a meta-analysis of the seven other GWAS samples (MAwo23andMe) and the Bonn sample (BN)

					MA			MAwo23andMe			23andMe			BN					
Chromosomal Region	lead SNP	Chr:Start -Stop	EA/OA	EAF	*P*-value	β	s.e.m	P-value	β	s.e.m	*P*-value	β	s.e.m	*P*-value	β	s.e.m	Consisteny of effect directions [1-yes, 0-no]	P<0.05 in MAwo23andMe	stronger effect size in BN
1p36.22	rs2095921	1:11,032,215-11,050,962	G/C	0.23	1.24×10^{-29}	-0.27	0.02	1.20×10^{-4}	-0.20	0.05	3.23×10^{-27}	-0.30	0.03	1.63×10^{-3}	-0.37	0.08	1	1	1
1p36.11	rs7534070	1:25,318,225-25,511,358	T/G	0.35	9.21×10^{-16}	-0.20	0.03	1.31×10^{-6}	-0.26	0.05	5.39×10^{-11}	-0.18	0.03	2.56×10^{-3}	-0.32	0.08	1	1	1
1p33	rs17371253	1:47,945,306-47,957,081	T/C	0.42	4.19×10^{-8}	0.12	0.02	0.16	0.08	0.06	9.16×10^{-8}	0.12	0.02	0.01	0.25	0.13	1	0	1
1p12	rs12083887	1:118,849,762-118,983,663	G/A	0.39	8.65×10^{-18}	-0.18	0.02	1.55×10^{-5}	-0.19	0.04	9.25×10^{-14}	-0.17	0.02	0.01	-0.26	0.08	1	1	1
1p12	rs17185996	1:119,203,617-119,789,289	T/C	0.13	9.96×10^{-15}	0.23	0.03	1.19×10^{-4}	0.25	0.06	1.62×10^{-11}	0.23	0.03	0.02	0.31	0.10	1	1	1
1q24.2	rs2421326	1:170,229,218-170,546,697	T/A	0.38	3.84×10^{-11}	0.14	0.02	4.23×10^{-3}	0.13	0.04	2.35×10^{-9}	0.14	0.02	2.73×10^{-3}	0.29	0.07	1	1	1
1q24.2	rs2206310	1:170,712,490-170,791,811	G/A	0.37	3.71×10^{-8}	0.12	0.02	0.03	0.12	0.06	4.40×10^{-7}	0.12	0.02	1.65×10^{-6}	0.47	0.16	1	1	1
2p25.2	rs12997617	2:6,458,933-6,573,741	T/C	0.14	5.82×10^{-12}	-0.20	0.03	4.45×10^{-10}	-0.42	0.07	3.60×10^{-6}	-0.15	0.03	1.26×10^{-4}	-0.63	0.09	1	1	1
2p23.1	rs9282858	2:31,609,942-33,040,343	T/C	0.03	8.85×10^{-18}	-0.51	0.06	5.49×10^{-4}	-0.49	0.14	1.48×10^{-15}	-0.51	0.06	0.06	-0.57	0.17	1	1	1
2p14	rs6546334	2:68,075,994-68,165,380	T/C	0.35	7.28×10^{-15}	-0.16	0.02	2.43×10^{-4}	-0.16	0.04	6.44×10^{-12}	-0.16	0.02	1.88×10^{-5}	-0.43	0.07	1	1	1
2q31.1	rs1819008	2:177,588,381-177,796,823	T/C	0.46	1.26×10^{-9}	-0.12	0.02	0.01	-0.12	0.05	3.70×10^{-8}	-0.12	0.02	0.06	-0.19	0.08	1	1	1
2q35	rs74333950	2:219,691,566-219,854,317	T/G	0.14	5.74×10^{-15}	-0.24	0.03	0.5	-0.05	0.08	2.24×10^{-16}	-0.27	0.03	2.44×10^{-3}	-0.42	0.21	1	0	1
2q37.3	rs11684254	2:239,638,300-239,764,032	G/C	0.34	1.08×10^{-39}	0.29	0.02	1.70×10^{-4}	0.22	0.06	2.46×10^{-37}	0.30	0.02	0.01	0.30	0.16	1	1	1
3q21.3	rs9850626	3:126,066,714-126,095,012	T/G	0.24	2.23×10^{-8}	-0.1	0.02	1.20×10^{-4}	-0.2	0.06	1.26×10^{-5}	-0.1	0.03	4.34×10^{-3}	-0.3	0.08	1	1	1

						3			1			2			1				
**3q23**	**rs10935316**	**3:139,020,298-139,032,333**	**G/C**	**0.20**	**4.09×10^{-11}**	**0.17**	**0.03**	**8.89×10^{-10}**	**0.37**	**0.06**	**1.01×10^{-5}**	**0.12**	**0.03**	**1.32×10^{-3}**	**0.43**	**0.09**	**1**	**1**	**1**
3q25.1	rs4679956	3:151,599,393-151,781,175	T/C	0.41	1.50×10^{-18}	0.18	0.02	0.09	-0.08	0.05	5.37×10^{-19}	-0.20	0.02	0.38	-0.08	0.10	1	0	0
4q21.21	rs982804	4:81,170,173-81,207,039	T/C	0.42	2.20×10^{-9}	-0.13	0.02	0.14	-0.10	0.07	5.75×10^{-9}	-0.13	0.02	0.01	-0.30	0.16	1	0	1
**4q24**	**rs142756290**	**4:105,851,807-105,903,045**	**G/C**	**0.07**	**3.81×10^{-8}**	**0.22**	**0.04**	**6.95×10^{-10}**	**0.44**	**0.07**	**1.17×10^{-2}**	**0.12**	**0.05**	**1.60×10^{-3}**	**0.60**	**0.10**	**1**	**1**	**1**
**4q25**	**rs145945174**	**4:107,507,213-107,888,788**	**G/A**	**0.07**	**1.26×10^{-13}**	**0.30**	**0.04**	**6.28×10^{-6}**	**0.37**	***0.08***	**2.12×10^{-9}**	**0.27**	**0.05**	**4.45×10^{-3}**	**0.72**	**0.12**	**1**	**1**	**1**
**4q32.3**	**rs17053607**	**4:168,717,680-168,789,572**	**G/A**	**0.05**	**1.06×10^{-8}**	**0.26**	**0.05**	**7.70×10^{-10}**	**0.62**	**0.10**	**9.76×10^{-4}**	**0.17**	**0.05**	**0.46**	**-0.21**	**0.34**	**1**	**1**	**1**
**5q13.3**	**rs141577316**	**5:73,425,638-73,479,375**	**T/C**	**0.02**	**5.71×10^{-10}**	**-0.58**	**0.09**	**1.27×10^{-9}**	**-0.88**	**0.15**	**2.50×10^{-3}**	**-0.37**	**0.12**	**0.03**	**-1.01**	**0.17**	**1**	**1**	**1**
5q33.3	rs6556350	5:157,844,535-157,880,002	T/C	0.47	3.17×10^{-08}	0.11	0.02	0.08	0.09	0.05	1.34×10^{-7}	0.12	0.02	0.11	0.15	0.11	1	0	1
**5q33.3**	**rs17643057**	**5:158,140,780-158,499,367**	**G/A**	**0.40**	**2.58×10^{-25}**	**-0.21**	**0.02**	**1.27×10^{-9}**	**-0.21**	**0.05**	**3.23×10^{-21}**	**-0.22**	**0.02**	**9.15×10^{-5}**	**-0.38**	**0.07**	**1**	**1**	**1**
6p25.3	rs12203592	6:396,321-421,281	T/C	0.17	2.00×10^{-11}	0.20	0.03	0.19	0.18	0.14	4.08×10^{-11}	0.20	0.03				1	0	-
**6p24.3**	**rs9380830**	**6:9,292,574-9,699,467**	**G/A**	**0.05**	**3.88×10^{-15}**	**-0.36**	**0.05**	**2.10×10^{-5}**	**-0.39**	**0.09**	**2.79×10^{-11}**	**-0.35**	**0.05**	**0.01**	**-0.60**	**0.13**	**1**	**1**	**1**
6p22.3	rs34061913	6:21,835,656-21,926,313	T/-	0.10	3.22×10^{-8}	-0.20	0.04	3.07×10^{-3}	-0.25	0.09	2.30×10^{-6}	-0.19	0.04	0.03	-0.43	0.13	1	1	1
6p21.1	rs4714811	6:44,639,723-44,676,420	T/C	0.19	3.43×10^{-10}	-0.16	0.03	0.01	-0.16	0.06	8.29×10^{-9}	-0.16	0.03	0.02	-0.29	0.09	1	1	1
**6q22.32**	**rs144578168**	**6:126,609,124-127,137,371**	**AAAAT/-**	**0.49**	**3.91×10^{-13}**	**-0.15**	**0.02**	**1.27×10^{-5}**	**-0.19**	**0.04**	**3.59×10^{-9}**	**-0.13**	**0.02**	**1.88×10^{-3}**	**-0.29**	**0.07**	**1**	**1**	**1**
7p21.1	rs6461387	7:18,765,520-18,933,411	G/C	0.40	7.92×10^{-33}	-0.25	0.02	0.53	-0.06	0.09	6.76×10^{-11}	-0.18	0.03	0.01	-0.34	0.19	1	0	1
7p12.3	rs12702262	7:46,846,164-46,948,331	G/C	0.24	2.94×10^{-8}	-0.14	0.02	0.23	-0.08	0.07	3.87×10^{-8}	-0.14	0.03	0.41	-0.09	0.12	1	0	0
**7q11.22**	**rs6952233**	**7:68,566,391-68,918,814**	**G/A**	**0.46**	**1.04×10^{-37}**	**-0.26**	**0.02**	**6.41×10^{-14}**	**-0.31**	**0.04**	**4.95×10^{-26}**	**-0.24**	**0.02**	**3.01×10^{-6}**	**-0.44**	**0.06**	**1**	**1**	**1**

8q23.1	rs74321310	8:108,847,459-109,469,932	G/A	0.01	4.25×10^{-10}	0.65	0.11	0.80	0.07	0.28	8.96×10^{-12}	0.75	0.11	0.99	-0.01	0.38	1	0	0
**9q32**	**rs201655553**	**9:116,511,364-116,691,072**	**AAAG/-**	**0.33**	**6.66×10^{-9}**	**0.14**	**0.02**	**3.57×10^{-7}**	**0.26**	**0.05**	**1.01×10^{-4}**	**0.10**	**0.03**	**1.85×10^{-3}**	**0.34**	**0.08**	**1**	**1**	**1**
**9q33.1**	**rs2416699**	**9:122,192,838-122,197,736**	**T/C**	**0.13**	**4.53×10^{-10}**	**0.18**	**0.03**	**8.82×10^{-15}**	**0.44**	**0.06**	**6.61×10^{-3}**	**0.09**	**0.03**	**0.07**	**0.27**	**0.11**	**1**	**1**	**1**
10q22.3	rs1907352	10:78,168,310-78,638,957	G/C	0.41	1.08×10^{-13}	0.15	0.02	9.87×10^{-3}	0.13	0.05	2.43×10^{-12}	0.16	0.02	0.07	0.17	0.08	1	1	1
10q26.13	rs3781458	10:126,278,648-126,537,000	T/C	0.38	3.31×10^{-13}	-0.15	0.02	0.04	-0.10	0.05	1.18×10^{-12}	-0.16	0.02	0.04	-0.20	0.08	1	1	1
**11p11.2**	**rs11037975**	**11:44,384,928-44,449,006**	**G/C**	**0.30**	**8.99×10^{-14}**	**-0.17**	**0.02**	**1.93×10^{-5}**	**-0.21**	**0.05**	**6.40×10^{-10}**	**-0.16**	**0.03**	**1.40×10^{-4}**	**-0.42**	**0.07**	**1**	**1**	**1**
12p12.1	rs9300169	12:26,438,616-26,446,289	G/A	0.27	1.59×10^{-13}	-0.17	0.02	8.33×10^{-3}	-0.15	0.06	4.43×10^{-12}	-0.18	0.03	0.08	-0.19	0.09	1	1	1
**12p11.22**	**rs10843026**	**12:27,986,524-28,102,722**	**G/A**	**0.05**	**3.37×10^{-9}**	**0.30**	**0.05**	**4.82×10^{-8}**	**0.55**	**0.10**	**2.24×10^{-4}**	**0.21**	**0.06**	**0.59**	**0.13**	**0.21**	**1**	**1**	**0**
12q13.12	rs4307773	12:50,878,227-51,319,795	T/C	0.41	1.47×10^{-9}	0.12	0.02	0.07	0.08	0.05	4.81×10^{-9}	0.13	0.02	0.51	0.06	0.09	1	0	0
**12q24.33**	**rs78472887**	**12:130,562,418-130,593,594**	**G/C**	**0.14**	**7.65×10^{-12}**	**0.20**	**0.03**	**9.13×10^{-6}**	**0.28**	**0.06**	**6.06×10^{-8}**	**0.18**	**0.03**	**0.05**	**0.28**	**0.11**	**1**	**1**	**1**
13q12.3	rs7997640	13:30,741,949-30,751,681	C/A	0.43	3.61×10^{-11}	-0.14	0.02	2.74×10^{-3}	-0.20	0.07	2.32×10^{-9}	-0.13	0.02	4.90×10^{-3}	-0.29	0.14	1	0	1
**15q12**	**rs4906800**	**15:26,120,550-26,121,173**	**T/A**	**0.07**	**9.29×10^{-9}**	**0.24**	**0.04**	**9.84×10^{-18}**	**1.07**	**0.12**	**2.53×10^{-3}**	**0.14**	**0.04**	**0.50**	**0.18**	**0.31**	**1**	**1**	**1**
**15q21.3**	**rs7173477**	**15:57,079,417-57,583,301**	**C/A**	**0.18**	**4.60×10^{-10}**	**-0.16**	**0.03**	**5.70×10^{-7}**	**-0.26**	**0.05**	**1.45×10^{-5}**	**-0.13**	**0.03**	**1.68×10^{-3}**	**-0.40**	**0.08**	**1**	**1**	**1**
15q22.2	rs17270216	15:60,836,794-60,842,258	G/A	0.22	1.38×10^{-8}	0.14	0.02	0.08	0.11	0.06	5.64×10^{-8}	0.15	0.03	0.58	0.07	0.14	1	0	0
**15q23**	**rs7182742**	**15:69,992,647-70,048,157**	**T/A**	**0.47**	**3.20×10^{-10}**	**-0.12**	**0.02**	**7.80×10^{-8}**	**-0.22**	**0.04**	**2.27×10^{-5}**	**-0.10**	**0.02**	**2.23×10^{-6}**	**-0.45**	**0.06**	**1**	**1**	**1**
16p13.12	rs246185	16:14,377,400-14,406,119	T/C	0.33	2.65×10^{-10}	0.14	0.02	8.52×10^{-3}	0.14	0.05	8.96×10^{-9}	0.14	0.02	0.09	0.23	0.11	1	1	1
**17q21.31**	**rs112550936**	**17:43,292,035-44,877,887**	**G/A**	**0.18**	**2.72×10^{-23}**	**-0.29**	**0.03**	**1.38×10^{-5}**	**-0.32**	**0.07**	**2.76×10^{-19}**	**-0.28**	**0.03**	**0.05**	**-0.30**	**0.11**	**1**	**1**	**1**
17q22	rs62060349	17:55,226,801-55,237,542	T/C	0.45	1.64×10^{-14}	-0.16	0.02	2.56×10^{-3}	-0.16	0.05	1.55×10^{-12}	-0.16	0.02	0.13	-0.14	0.11	1	1	0
**17q24.2**	**rs79806428**	**17:64,251,733-64,278,885**	**T/C**	**0.07**	**2.26×10^{-8}**	**-0.21**	**0.04**	**2.64×10^{-12}**	**-0.50**	**0.07**	**2.40×10^{-2}**	**-0.10**	**0.04**	**2.64×10^{-3}**	**-0.62**	**0.11**	**1**	**1**	**1**

**18q12.3**	**rs12606816**	**18:42,669,587-42,829,042**	**G/A**	**0.27**	**$9.36x10^{-21}$**	**-0.21**	**0.02**	**$4.86x10^{-8}$**	**-0.28**	**0.05**	**$8.98x10^{-15}$**	**-0.20**	**0.03**	**$1.84x10^{-3}$**	**-0.35**	**0.08**	**1**	**1**	**1**
**19q13.2**	**rs17318596**	**19:41,885,151-41,946,095**	**G/A**	**0.37**	**$1.67x10^{-8}$**	**0.12**	**0.02**	**$5.96x10^{-4}$**	**0.16**	**0.05**	**$4.41x10^{-6}$**	**0.11**	**0.02**	**0.04**	**0.20**	**0.08**	**1**	**1**	**1**
**20p12.2**	**rs733626**	**20:17,681,551-17,696,340**	**T/G**	**0.14**	**$4.14x10^{-9}$**	**0.17**	**0.03**	**$1.09x10^{-8}$**	**0.34**	**0.06**	**$3.95x10^{-4}$**	**0.12**	**0.03**	**0.87**	**0.02**	**0.14**	**1**	**1**	**1**
**20p11.22**	**rs148541866**	**20:21,365,463-21,517,472**	**T/G**	**0.01**	**$1.38x10^{-10}$**	**-0.58**	**0.09**	**$1.8x10^{-7}$**	**-0.72**	**0.14**	**$6.16x10^{-7}$**	**-0.48**	**0.12**	**0.21**	**-0.54**	**0.25**	**1**	**1**	**1**
**20p11.22**	**rs6113492**	**20:21,655,888-22,375,754**	**G/C**	**0.43**	**$1.23x10^{-162}$**	**0.51**	**0.02**	**$1.89x10^{-97}$**	**0.67**	**0.03**	**$3.34x10^{-77}$**	**0.43**	**0.02**	**$9.30x10^{-19}$**	**0.97**	**0.04**	**1**	**1**	**1**
20q12	rs6072223	20:39,616,593-39,647,516	T/A	0.16	$2.63x10^{-8}$	0.16	0.03	$1.06x10^{-3}$	0.21	0.06	$4.46x10^{-6}$	0.14	0.03	0.02	0.32	0.10	1	1	1
21q22.12	rs7280071	21:36,208,167-36,238,517	T/A	0.23	$9.19x10^{-11}$	-0.17	0.03	0.08	-0.13	0.07	$3.43x10^{-10}$	-0.17	0.03	0.04	-0.24	0.15	1	0	1
Xp22.31	rs5933688	X:8,880,680-8,917,206	G/A	0.29	$4.77x10^{-9}$	0.10	0.02	0.04	0.11	0.06	$3.73x10^{-8}$	0.10	0.02	0.01	0.20	0.09	1	1	1
Xp11.21	*rs185597083*	*X:54,875,521-55,133,845*	*C/A*	*0.04*	*$1.15x10^{-11}$*	*0.30*	*0.04*	*0.86*	*-0.03*	*0.18*	*$7.61x10^{-13}$*	*0.33*	*0.05*	*0.68*	*-0.08*	*0.23*	*0*	0	0
Xp11.21	rs143212632	X:55,775,616-56,492,197	G/A	0.04	$2.58x10^{-15}$	0.38	0.05	0.45	0.14	0.19	$3.10x10^{-16}$	0.39	0.05	0.62	0.11	0.20	1	0	0
Xp11.1	rs150535953	X:57,199,041-58,352,816	G/A	0.04	$1.39x10^{-17}$	-0.41	0.05	0.67	-0.23	0.52	$1.62x10^{-18}$	-0.41	0.05				1	0	-
**Xq12**	**rs2497911**	**X:62,340,191-67,813,682**	**C/A**	**0.19**	**$1.00x10^{-320}$**	**0.80**	**0.02**	**$6.85x10^{-158}$**	**1.01**	**0.04**	**$4.39x10^{-228}$**	***0.72***	**0.02**	**$1.66x10^{-19}$**	**1.12**	**0.04**	**1**	**1**	**1**
Xq26.3	rs4829906	X:136,618,815-136,957,253	G/A	0.40	$6.98x10^{-9}$	0.09	0.02	$6.88x10^{-3}$	0.12	0.04	$2.33x10^{-7}$	*0.08*	0.02	$4.44x10^{-3}$	0.19	0.06	1	1	1
																Number of SNP that fullfill criterion:	62	47 (**34**)	52

.

SNP - single nucleotide polymorphism; MPB - male-pattern baldness; EA -effect allele; OA - other allele; EAF- effect allele frequency; *P*-value - association *P*-value of the meta-analysis; β -effect size; s.e.m. - standard error of the mean; MA - association results from the MPB meta-analysis; 23andMe - association results of MPB risk variants in the 23andMe GWAS only; MAwo23andMe - association results from the meta-analysis of the seven remaining GWAS cohorts (BN, CoLaus, TwinUK, QIMR1, QIMR2, NBM, THISEAS), BN - association results from the clinically phenotyped BN cohort. Loci with an inconsistency in effect directions between 23andMe and MAwo23andMe are italicized. SNPs that show an association at P<0.05/63=7.9x10-4 in MAwo23andMe are printed in bold

Supplementary Table 9: Description of MPB credible SNP sets at the 63 genome-wide signficant risk loci for MPB.

Chromosomal Region	lead SNP	number of credible SNPs	genomic region/bp spanned [kb]
1p36.22	rs2095921	18	chr1:11,032,215-11,036,050 (3.84)
1p36.11	rs7534070	2	chr1:25,498,175-25,503,322 (5.15)
1p33	rs17371253	7	chr1:47,948,764-47,957,081 (8.32)
1p12	rs12083887	2	chr1:118,881,689-118,882,550 (0.86)
1p12	rs17185996	15	chr1:119,581,151-119,738,945 (157.79)
1q24.2	rs2421326	3	chr1:170,504,019-170,512,369 (8.35)
1q24.2	rs2206310	19	chr1:170,762,636-170,782,683 (20.05)
2p25.2	rs12997617	3	chr2: 6,551,033-6,556,781 (5.75)
2p23.1	rs9282858	1	
2p14	rs6546334	4	chr2:68,076,946-68,079,666 (2.72)
2q31.1	rs1819008	3	chr2:177,696,509-177,720,450 (23.94)
2q35	rs74333950	3	chr2:219,746,115-219,750,746 (4.63)
2q37.3	rs11684254	2	chr2:239,695,893-239,698,506 (2.61)
3q21.3	rs9850626	2	chr3:126,068,779-126,070,126 (1.35)
3q23	rs10935316	2	chr3:139,026,439-139,028,722 (2.28)
3q25.1	rs4679956	2	chr3:151,653,368-151,654,862 (1.49)
4q21.21	rs982804	1	
4q24	rs142756290	2	chr4:105,873,810-105,873,813 (0.003)
4q25	rs145945174	1	
4q32.3	rs17053607	3	chr4:168,779,959-168,789,572 (9.61)
5q13.3	rs141577316	1	
5q33.3	rs6556350	11	chr5:157,851,580-157,880,002 (28.42)
5q33.3	rs17643057	1	
6p25.3	rs12203592	1	
6p24.3	rs9380830	3	chr6:9,416,751-9,464,547 (47.80)
6p22.3	rs34061913	2	chr6:21,835,656-21,860,317 (24.661)

6p21.1	rs4714811	4	chr6: 44,649,758-44,659,586 (9.828)
6q22.32	rs144578168	8	chr6:126,678,268-127,000,954 (322.69)
7p21.1	rs6461387	3	chr7:18,897,511-18,903,015 (5.50)
7p12.3	rs12702262	13	chr7:46,879,550-46,943,759 (64.21)
7q11.22	rs6952233	6	chr7:68,567,586-68,598,231 (30.65)
8q23.1	rs74321310	3	chr8:109,102,918-109,469,932 (367.01)
9q32	rs201655553	2	chr9:116,555,661-116,565,926 (10.27)
9q33.1	rs2416699	3	chr9:122,195,880-122,196,740 (0.86)
10q22.3	rs1907352	7	chr10:78,192,757-78,290,960 (98.20)
10q26.13	rs3781458	5	chr10:126,337,060-126,355,129 (18.07)
11p11.2	rs11037975	1	
12p12.1	rs9300169	1	
12p11.22	rs10843026	2	chr12:28,093,612-28,093,691 (0.08)
12q13.12	rs4307773	15	chr12:51,144,432-51,221,127 (76.70)
12q24.33	rs78472887	5	chr12:130,562,418-130,568,656 (6.24)
13q12.3	rs7997640	1	
15q12	rs4906800	3	chr15:26,120,550-26,121,173 (0.62)
15q21.3	rs7173477	12	chr15:57,493,955-57,581,309 (87.35)
15q22.2	rs17270216	1	
15q23	rs7182742	10	chr15:69,992,647-70,044,227 (51.58)
16p13.12	rs246185	5	chr16:14,388,305-14,395,432 (7.13)
17q22	rs62060349	2	chr17:55,230,628-55,231,168 (0.54)
17q21.31	rs112550936	1	
17q24.2	rs79806428	1	
18q12.3	rs12606816	5	chr18:42,802,654-42,808,059 (5.41)
19q13.2	rs17318596	2	chr19:41,937,095-41,946,095 (9.00)
20p12.2	rs733626	2	chr20:17,685,345-17,685,349 (0.004)
20p11.22	rs148541866	1	
20p11.22	rs6113492	2	chr20:22,058,506-22,075,642 (17.14)
20q12	rs6072223	12	chr20:39,620,847-39,640,568 (19.72)

21q22.12	rs7280071	2	chr21:36,208,167-36,209,023 (0.86)
Xp22.31	rs5933688	14	chrX:8,880,680-8,917,206 (36.53)
Xp11.21	rs185597083	2	chrX:55,068,360-55,133,845 (65.49)
Xp11.21	rs143212632	2	chrX:56,197,395-56,291,380 (93.99)
Xp11.1	rs150535953	2	chrX:58,256,542-58,352,816 (96.27)
Xq12	rs2497911	1	
Xq26.3	rs4829906	3	chrX:136,951,523-136,957,253 (5.73)

kb – kilo bases

SUPPLEMENTARY REFERENCES

1. Brockschmidt, F.F. *et al.* Susceptibility variants on chromosome 7p21.1 suggest HDAC9 as a new candidate gene for male-pattern baldness. *Br J Dermatol* **165**, 1293-302 (2011).
2. Hillmer, A.M. *et al.* Susceptibility variants for male-pattern baldness on chromosome 20p11. *Nat Genet* **40**, 1279-81 (2008).
3. Firmann, M. *et al.* The CoLaus study: a population-based study to investigate the epidemiology and genetic determinants of cardiovascular risk factors and metabolic syndrome. *Bmc Cardiovascular Disorders* **8**(2008).
4. Sandhu, M.S. *et al.* LDL-cholesterol concentrations: a genome-wide association study. *Lancet* **371**, 483-491 (2008).
5. Andrew, T. *et al.* Are twins and singletons comparable? A study of disease-related and lifestyle characteristics in adult women. *Twin Res* **4**, 464-77 (2001).
6. Richards, J.B. *et al.* Male-pattern baldness susceptibility locus at 20p11. *Nat Genet* **40**, 1282-4 (2008).
7. Wetzels, J.F., Kiemeney, L.A., Swinkels, D.W., Willems, H.L. & den Heijer, M. Age- and gender-specific reference values of estimated GFR in Caucasians: the Nijmegen Biomedical Study. *Kidney Int* **72**, 632-7 (2007).

8. Gudmundsson, J. *et al.* Two variants on chromosome 17 confer prostate cancer risk, and the one in TCF2 protects against type 2 diabetes. *Nat Genet* **39**, 977-83 (2007).
9. Eriksson, N. *et al.* Web-based, participant-driven studies yield novel genetic associations for common traits. *PLoS Genet* **6**, e1000993 (2010).
10. Pickrell, J.K. *et al.* Detection and interpretation of shared genetic influences on 42 human traits. *Nat Genet* (2016).
11. Ellis, J.A., Stebbing, M. & Harrap, S.B. Genetic analysis of male pattern baldness and the 5alpha-reductase genes. *J Invest Dermatol* **110**, 849-53 (1998).
12. Dimitriou, M. *et al.* Exclusive olive oil consumption has a protective effect on coronary artery disease; overview of the THISEAS study. *Public Health Nutr* **19**, 1081-7 (2016).
13. Ripke, S. *et al.* Biological insights from 108 schizophrenia-associated genetic loci. *Nature* **511**, 421-+ (2014).
14. Heilmann, S. *et al.* Evidence for a polygenic contribution to androgenetic alopecia. *Br J Dermatol* **169**, 927-30 (2013).
15. Westra HJ*, et al.* Systematic identification of trans eQTLs as putative drivers of known disease associations. *Nat Genet* **45**, 1238-1243 (2013).
16. Consortium GT. Human genomics. The Genotype-Tissue Expression (GTEx) pilot analysis: multitissue gene regulation in humans. *Science* **348**, 648-660 (2015).
